# Ligand‐Ratio–Tuned DNA Nanocarriers Overcome Bladder Barriers for Ultralow‐Dose Intravesical Therapy

**DOI:** 10.1002/advs.78719

**Published:** 2026-11-09

**Authors:** Dunfang Liu, Xiao Chen, Hongliang Bao, Qian Xia, Gang Huang, Yang Yang

**Affiliations:** ^1^ Institute of Molecular Medicine and Shanghai Key Laboratory for Nucleic Acid Chemistry and Nanomedicine State Key Laboratory of Oncogenes and Related Genes School of Medicine Renji Hospital Shanghai Jiao Tong University Shanghai China; ^2^ Department of Nuclear Medicine School of Medicine Renji Hospital Shanghai Jiao Tong University Shanghai China

**Keywords:** DNA framework nanotechnology, GAG barrier penetration, intravesical drug delivery, ratio‐engineered nanotherapeutics, ultralow‐dose chemotherapy

## Abstract

Effective delivery to target cells across biological barriers requires coordinated integration of multiple surface functionalities, yet such integration remains difficult to control in nanoscale systems. This challenge is particularly evident in intravesical delivery, where selective tumor‐cell engagement must be coupled with efficient transport through the glycosaminoglycan/mucus‐associated luminal environment. Here we present a ratio‐tunable DNA framework drug carrier (dFDC) that enables precise control over two orthogonal surface modules: an Sgc8 aptamer for targeting and a fluorocarbon (F–C) chain for barrier transit. By programming their valency across 30 external sites of an icosahedral framework, we identify a nonlinear cooperative regime in which transport, uptake, and cytotoxicity are jointly optimized. Performance is governed by the relative proportion of functional modules rather than their absolute density. At the optimal Sgc8/fluorocarbon configuration, the carrier enhances diffusion in chondroitin sulfate, improves transport across a GAG‐associated epithelial barrier model, and increases tumor‐cell killing after barrier transit. In orthotopic models, this dFDC achieves complete tumor remission at ∼1/1500 of the clinical gemcitabine dose with no recurrence over 60 days. This work introduces ligand‐ratio engineering as a general strategy for balancing orthogonal transport requirements across hierarchical biological barriers.

## Graphical abstract

A programmable DNA framework drug carrier coordinates barrier transport, tumor targeting, and drug delivery through ligand‐ratio engineering. Systematic exploration of the Sgc8/fluorocarbon design space reveals a nonlinear optimal balance between penetration and cellular engagement, translating multifunctional integration into durable bladder tumor remission at an ultralow intravesical gemcitabine dose.

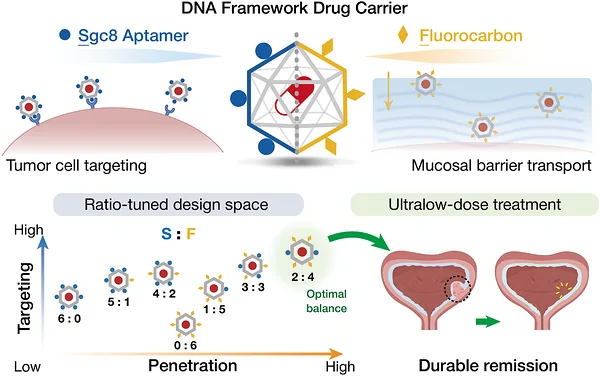

## 1 Introduction

In mucosal drug delivery, penetration and targeting impose distinct functional demands that are often difficult to coordinate within a single nanoscale system. This trade‐off is especially pronounced in the bladder, where the dense glycosaminoglycan layer and dynamic urine flow severely limit drug transport. Bladder cancer is one of the most recurrent malignancies of the urinary tract, and intravesical drug delivery (IDD) remains the standard adjuvant therapy after transurethral resection [1]. Despite its advantage of direct lesion exposure and minimized systemic toxicity, IDD efficacy is severely constrained by the multilayered barriers of the bladder wall. The glycosaminoglycan (GAG) layer—a 10–20 µm hydrophilic mucinous coating enriched in chondroitin and heparan sulfate—forms the first and most formidable obstacle, limiting diffusion and trapping cationic or hydrophobic agents through nonspecific interactions [2, 3]. Beneath this barrier, dense extracellular matrix and heterogeneous tumor cell populations further restrict drug access and retention [4]. In addition, dynamic urine flushing shortens residence time, so even high‐dose intravesical gemcitabine [5] or mitomycin C [6] achieves only partial remission with frequent recurrence, while dose escalation exacerbates local toxicity rather than improving benefits [7, 8].

Nanotechnological strategies have attempted to overcome individual barriers—fluorinated chitosan [9] and urease‐powered silica [10] to reduce mucin adhesion and improve GAG penetration [11], or aptamer/antibody‐modified carriers [12, 13] for receptor‐specific tumor targeting. However, these strategies generally optimize a single dimension [14, 15]. Effective IDD requires the coordinated execution of two functions: (i) navigating the GAG/mucus‐associated luminal environment to access tumor‐exposed regions, and (ii) selectively engaging accessible tumor cells for efficient uptake. Achieving both simultaneously remains a central unmet challenge. Instead of relying on the misconception of functional additivity—assuming that penetration enhancers and targeting ligands will simply contribute independent benefits—effective design requires achieving precise stoichiometric balance rather than component accumulation. DNA nanotechnology offers a unique solution [16, 17]. Owing to its addressable architecture, a DNA framework can precisely control the number, spatial pattern, and ratio of surface ligands [18]. Such programmable valency has enabled polyhedral DNA structures to present multivalent antigens for vaccines [19, 20] and to fine‐tune ligand density for targeted nucleic‐acid delivery [21, 22], demonstrating that DNA frameworks are inherently well suited for engineering tunable multifunctional nanomedicines.

Here we introduce a DNA framework drug carrier (dFDC) engineered to integrate two biomechanically orthogonal modules: a fluorocarbon (F‐C) chain that improves transport through the GAG/mucus‐associated luminal environment by reducing mucin adhesion [23, 24, 25], and an Sgc8 aptamer that enables high‐affinity engagement of PTK7‐positive bladder tumor cells [26] (Figure 1A). Using the digitally addressable architecture of a 43‐nm DNA icosahedron, we created a ratio‐tuned library that systematically maps how module valency affects transport, binding, and cytotoxicity. Microscale thermophoresis and transwell infiltration assays revealed that balanced Sgc8:(F‐C) ratios generate cooperative gains in diffusivity and barrier penetration, illustrating a design balance rarely quantified in intravesical delivery. In orthotopic mouse models, the optimized dFDC configuration—equipping 1/3 Sgc8 and 2/3 F‐C modules—achieved complete tumor remission (100% CR) after only two instillations, using ∼1/1500 of the clinical gemcitabine dose [27], with no recurrence over 60 days. This ratio‐tuned DNA framework establishes a biomolecular engineering paradigm for ultralow‐dose, high‐precision intravesical chemotherapy.

**FIGURE 1:**
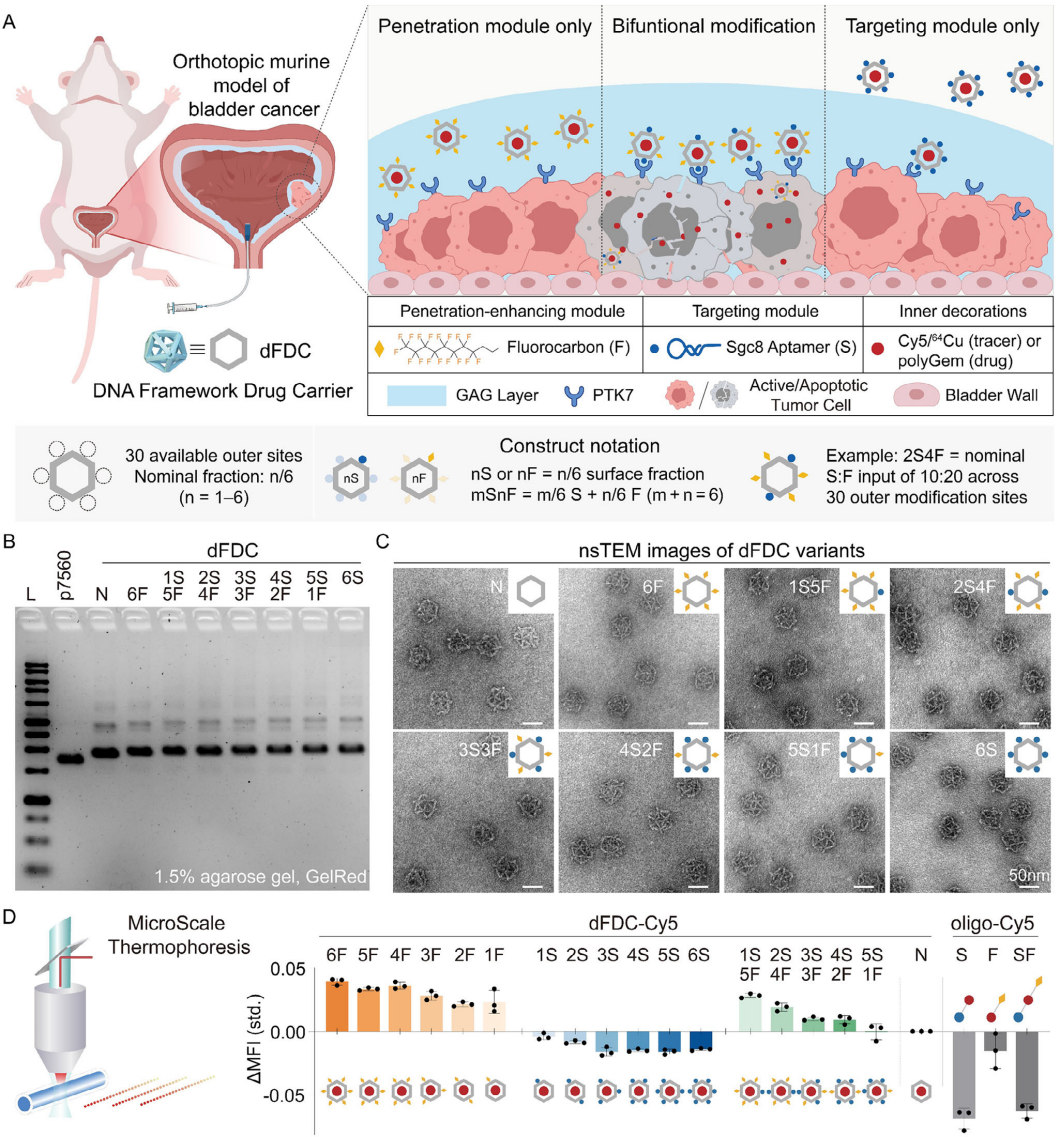
Conceptual design, modification strategy, and structural characterization of dFDC. (A) Schematic overview of the ratio‐engineered DNA framework drug carrier (dFDC), showing its functional components and intended use in an orthotopic murine bladder‐cancer model. Surface positions are schematic; substoichiometric modification represents probabilistic occupation of the common outer‐handle pool. (B) Native agarose gel electrophoresis and (C) negative‐stain TEM images of dFDC variants bearing saturated outer‐surface modifications. Scale bar: 50 nm. (D) Microscale thermophoresis analysis revealing ratio‐dependent motional behavior of Cy5‐labeled dFDC variants and oligo controls in a chondroitin sulfate–supplemented viscous medium.

## 2 Results

### 2.1 Construction, Ratio Engineering, and Motional Characterization of dFDCs

To establish a programmable platform for intravesical delivery, we constructed a 43‐nm DNA icosahedron comprising rigid four‐helix‐bundle edges and a geometrically insulated inner cavity. The architecture contains 60 internal handles for high‐density cargo loading and 30 external handles for modular surface functionalization (Figure S1). This separation of inner/outer topology enables fully independent control over drug payload and ligand valency.

Onto the external handles, we installed two classes of functional strands: a fluorocarbon‐modified oligo (F) to promote transport across the mucus‐rich GAG layer, and an Sgc8 aptamer (S) to target the PTK7 receptor on bladder cancer cells. The choice of Sgc8 was further supported by clinical evidence showing elevated PTK7 expression in a substantial subset of human bladder cancers relative to adjacent normal urothelium, together with recent first‐in‐human validation of intravesical Sgc8‐based PET imaging [28]. We supplied S and F at predefined chemical ratios spanning 0/6 to 6/6, corresponding to probabilistic occupation of the 30 available outer handles (Figure S1). Thus, the resulting nS, nF, and mSnF constructs represent ensemble‐average surface valencies rather than predefined positional patterns. This strategy generated a systematic S–F modification library, including single‐modified *n*S or *n*F (*n* = 1–6) and dual‐modified *m*S*n*F constructs (*m*+*n* = 6), allowing quantitative analysis of how species identity, surface density, and S:F ratio influence dFDC behavior.

All modified structures maintained monodispersity and intact icosahedral morphology, as confirmed by sharp agarose gel electrophoresis (AGE) bands (Figure 1B) and negative‐stain transmission electron microscopy (nsTEM) imaging across the entire S/F series (Figure 1C and Figure S2). Migration patterns on AGE further reported the expected hydrophobicity trends and molecular weight changes: increasing F‐content slightly accelerated electrophoretic mobility, whereas increasing S‐content produced marked retardation (Figure S3). To experimentally validate the programmability of the common outer‐handle pool, we further performed dual‐fluorescence calibration using AF488‐ and AF647‐labeled anti‐z strands. dFDC‐associated fluorescence increased linearly with the nominal input fraction for each tracer, while complementary dual‐color inputs produced reciprocal fluorescence changes across the 1:5–5:1 ratio series, supporting predictable ratio‐programmable co‐occupation of the outer handles (Figure S4). We similarly evaluated the loading behavior of the 60 inward‐facing handles using a Cy5‐labeled surrogate cargo strand. Framework‐associated fluorescence increased nearly linearly over 0–60 input equivalents, supporting quantitatively programmable inner‐handle loading; accordingly, 60 equivalents were used as the full‐loading condition in subsequent experiments (Figure S5). To distinguish framework‐dependent phenomena from those originating solely from the functional modules, we also prepared monovalent oligo controls—Cy5‐S, Cy5‐F, and the bifunctional Cy5‐SF—via denoted hybridization (sequences in Table S1). PAGE analysis confirmed their successful assembly (Figure S6). These controls serve as essential references for distinguishing framework‐enabled effects from intrinsic ligand properties in later assays.

To determine how surface stoichiometry influences nanoscale motional behavior in a polymer‐rich viscous medium, we measured the thermophoretic responses of each dFDC variant using microscale thermophoresis (MST) in chondroitin sulfate–supplemented buffer (1.188 mg/mL) (Figure S7). Using unmodified dFDC‐N as the baseline, the direction and magnitude of the terminal ΔMFI provided an intuitive indicator of relative mobility: a more positive ΔMFI reflected faster thermophoretic displacement, whereas a negative ΔMFI indicated slower motion. Across the S–F series, fluorocarbon‐bearing dFDCs consistently displayed more positive ΔMFI values than dFDC‐N, indicating enhanced mobility attributable to reduced polymer entanglement and diminished adhesive drag. In contrast, increasing proportions of Sgc8 aptamers produced progressively more negative ΔMFI signals, consistent with the expected mobility reduction from larger, more charged, and highly interactive aptamer coronas. Mixed S/F displays showed intermediate yet generally faster motion relative to dFDC‐N, underscoring the dominant contribution of fluorocarbon chains to enhancing molecular transport (Figure 1D). Compared with the framework‐based constructs, all oligo–Cy5 controls migrated substantially more slowly, and the presence of a fluorocarbon unit alone could not compensate for this reduction. This contrast highlights a key material property: the isotropic, rigid, and well‐defined geometry of the DNA icosahedron intrinsically improves diffusional behavior, serving as a transport‐advantaged scaffold onto which functional modules exert ratio‐dependent modulation.

### 2.2 Ratio‐Governed Mucus Penetration and Cytotoxic Response of Modified dFDCs

To determine whether the ratio‐dependent motional behaviors observed in polymer‐rich media translate into enhanced barrier traversal, we employed a transwell system in which RT4 cells were pre‐cultured on the insert membrane to establish a cell‐derived GAG‐associated epithelial barrier [29], with functional barrier establishment supported by TEER stabilization by Day 2 (Figure S8). Upon adding Cy5‐labeled dFDC variants to the upper chamber, the fluorescence appearing in the lower chamber provided a quantitative measure of transport across this cell‐derived barrier.

All constructs displayed a characteristic two‐phase permeation profile (Figure S9). During the initial 0–1 h interval (Stage 1), samples exhibited a rapid but non‐discriminative entry phase, consistent with transient concentration‐gradient–driven redistribution across the donor–barrier–receiver system. After ∼1 h, permeation transitioned into a slower, approximately linear regime (Stage 2), in which construct‐dependent differences became highly reproducible. We therefore used the Stage‐2 slopes to quantitatively compare the relative barrier‐translocation behavior of the different surface compositions (Figure S9). Using these linear‐region permeation rates, we next quantified how surface composition shapes transport across the GAG‐associated epithelial barrier (Figure 2A). Within each modification series, clear composition‐dependent trends emerged. In the S‐only group, translocation efficiency decreased progressively with increasing Sgc8 valency, consistent with stronger interactions with the GAG‐associated epithelial interface and reduced passage into the lower compartment (Figure S10). In contrast, F‐bearing constructs—both F‐only and S/F dual‐modified variants—showed uniformly elevated permeation, supporting a role for fluorocarbon motifs in enhancing transport across the GAG‐associated epithelial barrier. Notably, permeation did not increase monotonically with F density: across both the F‐only and SF series, constructs carrying 3/6–4/6 F modules (≈15–20 F strands per particle) exhibited the highest steady‐state flux, whereas constructs with either lower or fully saturated F content displayed reduced rates. This identifies a ratio‐dependent optimal regime in which fluorocarbon‐mediated transport enhancement is maximized. Compared with the strongly F‐dependent permeation of the modified dFDCs, all oligo–Cy5 controls exhibited uniformly low translocation, closely matching their reduced molecular mobility in the MST assay and underscoring the consistent relationship between nanoscale motional behavior and diffusion‐driven barrier permeation.

**FIGURE 2:**
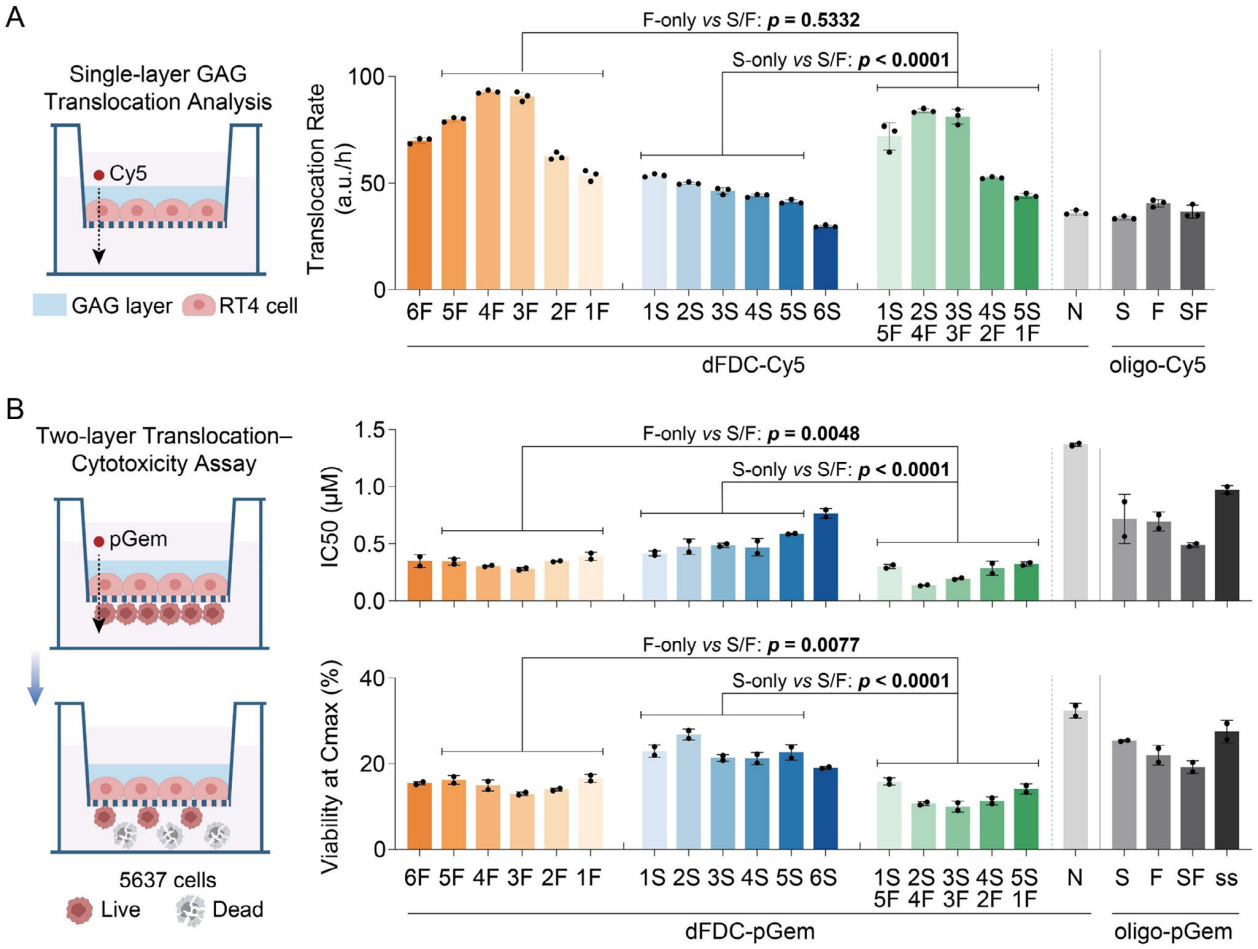
Ratio‐governed mucus penetration and cytotoxic response of modified dFDCs. (A) Schematic and transwell‐based assessment of dFDC transport across an RT4 cell‐derived GAG‐associated epithelial barrier. Fluorescence increment rates in the bottom chamber during the Stage 2 (1–6 h; Figure S9) are used to compare translocation performance. (B) Cytotoxicity of gemcitabine‐loaded dFDC variants evaluated in a two‐layer transwell model. Flow cytometry of the bottom‐layer 5637 cells across a concentration series was used to generate dose–response curves (Extended Data S1). For series‐level comparisons, formulations were aligned according to the corresponding S/F composition axis. The S/F‐dual series (1S5F–5S1F) was compared with the matched F‐only series (5F–1F) and S‐only series (1S–5S) using two‐way ANOVA.

To quantify the cytotoxic effect of gemcitabine delivered after crossing the mucus barrier, we used a two‐layer transwell model in which RT4 cells were cultured on the upper surface of the insert membrane, while GFP‐5637 bladder tumor cells were seeded on the opposite side of the membrane and immersed in the bottom chamber (Figure 2B). Gemcitabine was incorporated into dFDCs as a 20‐nt polymeric form (pGem) covalently attached to the 60 internal anti‐handle strands. After 6 h of exposure, flow cytometry of the bottom‐layer 5637 cells across a concentration series yielded dose–response curves (Extended Data S1), from which IC_50_ values, viability at C_max_ (Figure 2B), as well as the corresponding area‐under‐curve (AUC, see Figure S11) metrics were calculated.

The unmodified dFDC‐N showed only limited cytotoxic activity, whereas adding either penetration or targeting modules resulted in clear increases in downstream killing. At the IC_50_ level, single‐modified variants displayed trends that closely paralleled their permeation efficiencies (Figure 2A). In the C_max_ and AUC metrics, S‐only constructs exhibited gradually improved efficacy with increasing S valency, indicating a modest dose‐dependent benefit of high aptamer loading. By contrast, the dual‐modified dFDCs containing 3/6–4/6 F modules paired with moderate S content (3S3F and 2S4F) achieved the strongest post‐translocation killing, integrating low IC_50_, high C_max_‐mediated cytotoxicity, and high AUC into a unified performance profile. Importantly, these constructs possessed permeation rates nearly identical to those of F‐only variants with matching F densities, yet their cytotoxic activities were substantially higher. This divergence underscores a clear synergistic interaction between the two functional modules, in which fluorocarbon‐mediated barrier traversal is complemented by Sgc8‐mediated tumor‐cell engagement to amplify downstream drug action. In equivalent‐dose oligo‐pGem controls, the dual‐modified SF oligo showed only modest improvement relative to single‐modified oligos.

To complement the barrier‐dependent transwell assay and isolate the contribution of intrinsic cellular interactions, we evaluated uptake, intracellular trafficking, and direct cytotoxicity of the dFDC variants under equal‐dose direct incubation. Flow‐cytometry uptake measurements (Figure S12) showed that, in the absence of a mucus barrier, S‐bearing constructs dominated internalization, with F also contributing above the unmodified scaffold, and S/F‐dual‐modified variants maintaining a ratio‐dependent synergistic advantage. Confocal colocalization analysis (Figure S13) further revealed that increasing S valency strengthened lysosomal routing, whereas constructs containing ∼4/6 F modules displayed markedly reduced lysosomal colocalization. This behavior, consistent with our prior observations [30] and supported by reports that cytosolic processing of pGem yields superior metabolic efficacy [31], suggests that enhanced uptake coupled with partial lysosomal avoidance represents an optimal design target, achieved through balanced S/F presentation.

Direct cytotoxicity assays (Extended Data S2 and Figure S14) showed only modest differences within single‐module series at the IC_50_ level, yet dual‐modified constructs again outperformed their single‐modified counterparts, with 2S4F displaying the lowest IC_50_ and strongest C_max_‐associated killing. This indicates that the same S/F synergy observed in the transwell assay persists when barrier effects are removed. Finally, PTK7 expression profiling (Figure S15) confirmed substantial overexpression in bladder cancer cells (5637 cells) relative to normal urothelial cells (SV‐HUC‐1 cells), and viability assays (Figure S16) demonstrated that dFDC‐2S4F achieves high potency against tumor cells while maintaining low toxicity toward SV‐HUC‐1, supporting its favorable balance between efficacy and safety.

Beyond cytotoxicity and selectivity, we also assessed whether the DNA framework itself elicited an innate DNA‐sensing response [32]. RNA‐seq analysis of cells treated with N, 6S, 6F, 4S2F, 3S3F, and 2S4F showed no coordinated induction of core cGAS–STING pathway genes (CGAS, STING1, TBK1, and IRF3) or representative downstream interferon‐stimulated genes, including in the unmodified framework and optimized 2S4F groups (Figure S17). These data indicate no prominent cGAS–STING‐associated transcriptional response under the conditions examined, although transient or post‐translational pathway activation cannot be excluded.

### 2.3 In Vivo Tumor Targeting and Retention of Ratio‐Optimized dFDCs

Having confirmed the superior GAG ‐associated epithelial barrier traversal and cellular delivery properties of balanced S/F presentations in vitro, we next investigated whether these advantages translated to improved behavior in the dynamic urinary environment (Figure 3A). Before radiolabeling, we first verified that both the outer and inner functional strands remained stably associated with the framework in urine. Agarose gel electrophoresis showed that both outer and inner strands remained well associated with the framework during the first hour in freshly collected mouse urine, corresponding to the intravesical treatment window, although the inner‐strand fluorescence gradually decreased upon prolonged incubation at 4–6 h (Figure S18).

**FIGURE 3:**
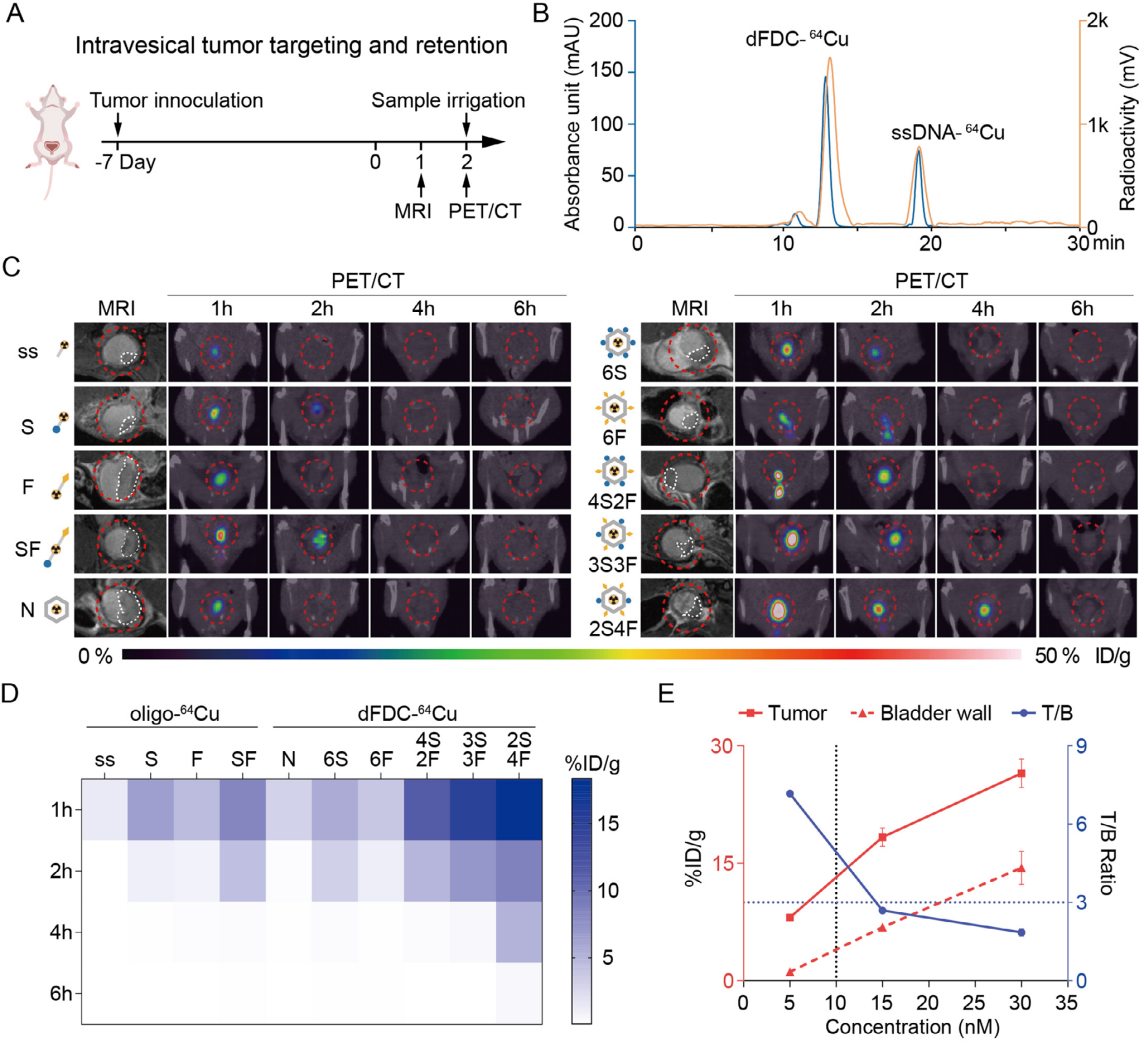
In vivo intravesical tumor targeting and retention of ratio‐engineered dFDCs. (A) Schematic of the orthotopic bladder tumor model and intravesical administration timeline for MRI and PET/CT imaging. (B) Radio–HPLC characterization of ^64^Cu‐labeled dFDCs and ssDNA controls showing radiochemical purity and elution profiles. (C) Representative MRI and serial PET/CT images (1–6 h post‐instillation) of mice treated intravesically with oligo‐^64^Cu controls and dFDC‐^64^Cu variants. Bladders are outlined with red dashed circles, and tumor regions in the MRI images are outlined with white dashed contours. (D) Heat map of bladder tumor‐associated radioactivity (%ID/g) at different time points for oligo‐^64^Cu and dFDC‐^64^Cu groups. (E) Quantitative analysis of tumor uptake, bladder‐wall retention, and tumor‐to‐bladder ratio (T/B) for dFDC‐2S4F at increasing intravesical doses (2 mice/group).

To visualize their in vivo distribution, individual constructs were labeled with ^64^Cu chelated NOTA–ssDNA. Radiochemical purity was confirmed by radio–TLC and radio–HPLC (Figure 3B and Figure S19), enabling reliable quantification of particle retention and tumor association using PET/CT imaging. Animals were randomized before administration to minimize intergroup differences in baseline tumor burden. Mice bearing orthotopic 5637 tumors received each ^64^Cu‐labeled formulation intravesically, followed by bladder irrigation and serial PET/CT scans up to 6 h post‐instillation (Figure 3C and Figure S20). In an independent validation cohort bearing luciferase‐expressing orthotopic tumors, baseline bioluminescence quantification further confirmed comparable tumor burdens across all formulation groups (Figure S21).

A global comparison of tumor‐associated radioactivity is summarized in the heatmap (Figure 3D), which provides an overview of retention levels across oligo controls and all ratio‐engineered dFDC variants. Consistent with the in vitro transport hierarchy, dual‐modified constructs showed markedly higher intravesical retention and tumor accumulation than single‐module or unmodified controls. Pure aptamer (6S) or pure fluorocarbon (6F) carriers exhibited only transient bladder signals that rapidly declined after 1–2 h, whereas mixed S/F ratios maintained significantly stronger tumor‐localized radioactivity. Among all variants, 2S4F displayed the highest tumor uptake at 1 h and the slowest subsequent decay, indicating that the optimized S/F balance enables both rapid access to the tumor surface and prolonged local engagement. Constructs with suboptimal ratios (4S2F, 3S3F) showed intermediate retention, whereas oligo‐^64^Cu controls and bare framework (N) exhibited near‐complete loss of signal by 2 h, confirming that neither DNA nanostructure geometry nor ligand identity alone is sufficient for intravesical persistence. Consistent with the radiotracer measurements, ex vivo Cy5 imaging in this baseline‐comparable cohort reproduced the overall composition‐dependent retention trend (Figure S21).

To determine a therapeutically relevant dosing window, we next instilled ^64^Cu–2S4F at increasing concentrations and quantified tumor and bladder‐wall uptake (Figure 3E). Both tumor (T) and bladder‐wall (B) accumulation (%ID/g) rose with dose, but because the increase in nonspecific bladder retention paralleled that in tumor uptake, the T/B ratio decreased progressively across the examined range. In intravesical radiotracer studies, a T/B ratio >3 is commonly considered indicative of meaningful tumor selectivity [33]. Notably, the tumor and bladder‐wall curves intersected at ∼11 nM, the point at which T/B remained above 3 while maintaining adequate absolute tumor uptake. We therefore selected 10 nM as the optimal intravesical dosing concentration, achieving a practical balance between maximizing tumor accumulation and limiting off‐target bladder‐wall retention, and providing an evidence‐based parameter for the dosing regimen used in subsequent therapeutic studies.

### 2.4 In Vivo Therapeutic Efficacy of Ratio‐Engineered dFDCs

To evaluate whether ratio‐engineered dFDCs enhance intravesical chemotherapy in vivo, we established an orthotopic bladder tumor model using 5637‐luc cells, a human bladder carcinoma line stably expressing luciferase to permit quantitative bioluminescence tracking of tumor burden. Mice received seven intravesical instillations—every five days on Days 0, 5, 10, 15, 20, 25, and 30—as outlined in Figure 4A. The dFDC dosing concentration (10 nm in 100 µL) was selected based on the in vivo T/B analysis as discussed in Figure 3E. To increase the per‐particle drug payload, each dFDC carried 60 copies of 40‐nt pGem, resulting in a monomer‐equivalent gemcitabine dose of 2.4 µM (∼6.3 µg/mL) per instillation. For rigorous comparison, oligo‐pGem and the low‐dose free‐gemcitabine (LD) group were administered at the same gemcitabine‐monomer equivalent. In addition, a high‐dose free‐gemcitabine (HD) group was included following clinically reported intravesical concentrations of 10 mg/mL, corresponding to ∼1,578‐fold higher monomer dose than LD.

**FIGURE 4:**
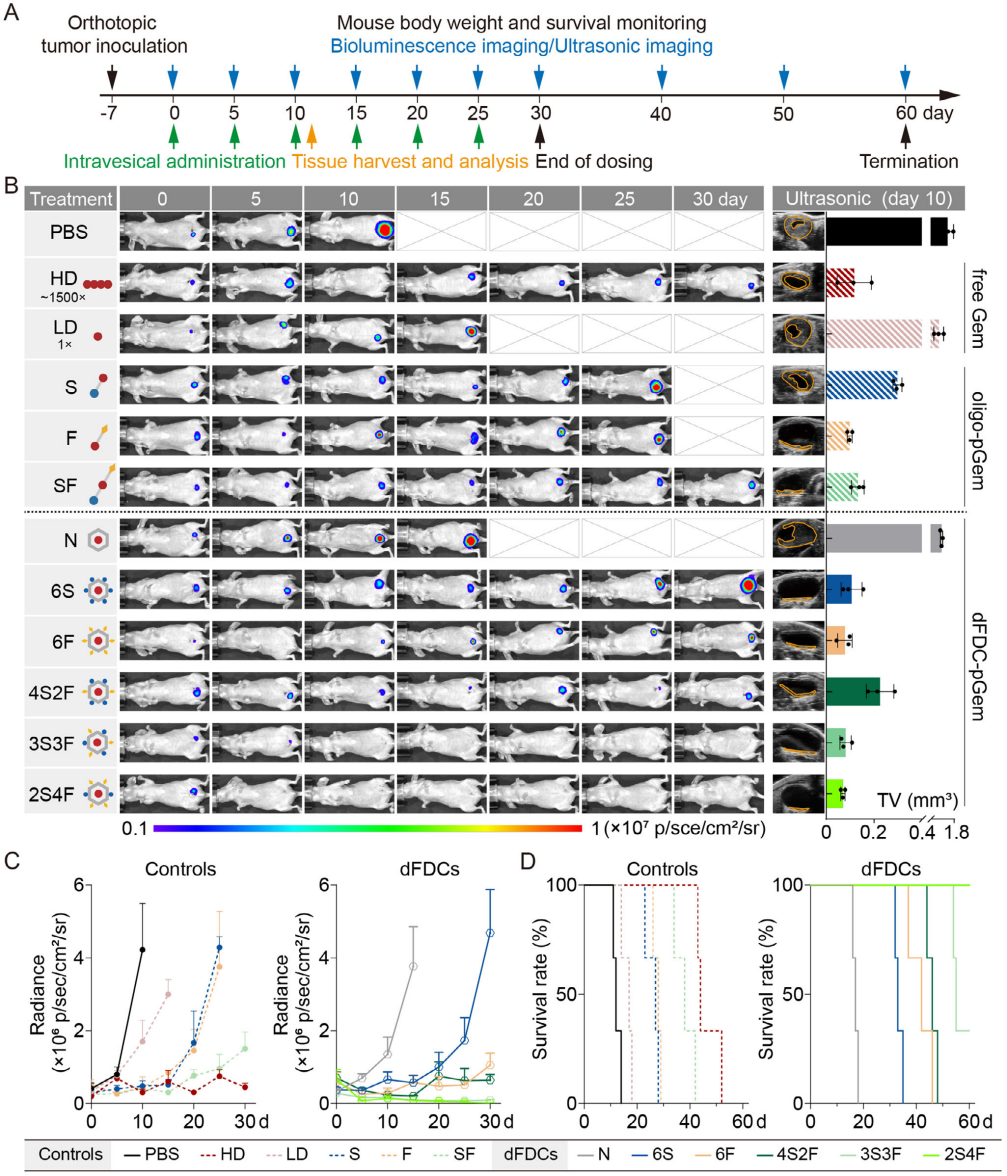
Intravesical therapeutic evaluation of dFDC‐pGem formulations in an orthotopic bladder cancer model. (A) Timeline of the therapeutic study. Mice bearing orthotopic 5637‐luc bladder tumors received intravesical treatments on the indicated days, followed by longitudinal monitoring of body weight, survival, bioluminescence signals, and ultrasonic imaging. (B) Bioluminescence images of bladder tumors at the indicated time points (0–30 days) after intravesical administration of the listed formulations. Ultrasonic images acquired on Day 10 are shown to the right, with tumor regions outlined by orange contours and the corresponding tumor volumes summarized in the adjacent bar graph. (C) Quantification of tumor radiance (photons·s^−^^1^·cm^−^^2^·sr^−^^1^) for controls (left) and dFDC‐pGem formulations (right). Curves represent mean ± SD. for each treatment group. (D) Survival curves over the 60‐day study for mice receiving control formulations (left) or dFDC‐pGem variants (right). Each treatment group contained three animals.

During the 30‐day dosing phase, longitudinal bioluminescence and radiance quantification revealed distinct response patterns across the control formulations (Figure 4B,C and Figures S22 and S23). PBS and LD free gemcitabine provided virtually no therapeutic benefit, with all mice succumbing to rapidly expanding tumors within 12 and 19 days, respectively. Single‐modified oligo‐pGem‐S or ‐F afforded only modest benefit, delaying overt tumor outgrowth and death by roughly 10–15 days relative to LD, but ultimately failing to alter the overall trajectory of progression. By contrast, HD free gemcitabine and the dual‐modified oligo‐SF control achieved a marked suppression of tumor expansion in all animals during the 0–30 day period, indicating that either a very high monomer dose or combined S/F chemistry can transiently restrain tumor growth even in the absence of a framework carrier.

Among dFDC‐based pGem formulations, dFDC‐N behaved similarly to LD free gemcitabine, with all animals reaching humane endpoints before Day 20 despite the presence of the framework. Incorporation of S alone (6S‐pGem) modestly extended survival to the end of the dosing phase, but radiance signals began to rise steeply after Day 20, indicating limited control of late‐stage growth. In contrast, F‐containing carriers—including 6F, which lacks S entirely—produced a more sustained slowing of tumor expansion during the dosing period, consistent with the role of fluorocarbon modules in facilitating mucosal transport within the intravesical environment. 4S2F‐pGem performed comparably to 6F‐pGem, indicating that limited F loading—rather than the level of S—constrains early therapeutic activity. Ratio‐optimized dual‐modified constructs, however, produced a clear shift in therapeutic potency: 3S3F‐ and 2S4F‐pGem reduced bioluminescence to nearly undetectable levels by Day 10 in all mice, representing a marked transition from growth suppression to near‐complete tumor clearance during the dosing window. Ultrasonography examinations [34] corroborated these bioluminescence findings across the full imaging period (Figures S24 and S25). Accordingly, Day‐10 ultrasound images and tumor‐volume measurements (Figure 4B, right) were selected as a unified evaluation time point at which all animals remained alive and treatment‐dependent differences were most clearly resolved. The resulting pattern of in vivo tumor control closely mirrored the transwell killing hierarchy, reinforcing that balanced S/F ratios are essential for converting enhanced barrier traversal and cellular uptake into maximal therapeutic benefit.

To further examine the mechanism underlying this early therapeutic response, an additional parallel cohort was treated using the same regimen and tumors were harvested on Day 11, after three intravesical administrations, for apoptosis‐associated histological analysis. Cleaved caspase‐3 immunostaining and TUNEL staining showed minimal apoptotic signals in PBS and LD controls, intermediate responses in the less effective formulations, and substantially stronger positivity in the optimized 3S3F and 2S4F groups, with 2S4F showing the highest overall apoptotic response (Figure S26). These findings support enhanced apoptotic tumor‐cell death in the ratio‐optimized formulations and provide histological evidence consistent with the early tumor‐suppression profiles observed by bioluminescence and ultrasonography.

Longitudinal follow‐up further revealed marked differences in response durability after treatment cessation on Day 30. HD free gemcitabine, 6F‐pGem, and 4S2F‐pGem—each of which only marginally slowed tumor growth during treatment—exhibited rapid tumor regrowth once dosing stopped, resulting in uniform mortality shortly after the end of the dosing window (Figures S22–S25). In contrast, 3S3F‐ and 2S4F‐pGem maintained tumor suppression beyond the treatment phase, reflecting their markedly stronger antitumor activity. However, all mice treated with 3S3F‐pGem eventually displayed bioluminescence recurrence following treatment withdrawal, indicating incomplete durability of the response. Only the 2S4F‐pGem group remained fully tumor‐free through Day 60, with 100% survival and no detectable recurrence in any animal. Survival analysis corroborated these trends, clearly separating the transient, intermediate, and durable response profiles across formulations (Figure 4D). Terminal bladder histology further corroborated the longitudinal imaging results. In less‐effective treatment groups, residual or recurrent tumor tissue remained evident at the respective terminal endpoints, whereas 2S4F‐treated bladders collected on Day 60 showed preserved architecture with no histologically identifiable residual tumor in the examined sections (Figures S27 and S28).

Throughout the 30‐day treatment and follow‐up period, body‐weight fluctuations closely paralleled tumor burden across all groups, showing no systematic differences attributable to any formulation (Figure S29). Histopathological examination of major organs collected from tumor‐bearing mice after three instillations revealed no pathological alterations associated with dFDCs, their S/F modifications, or any form of gemcitabine administration—including the 10 mg/mL high‐dose free drug (Figure S30). To further assess local and systemic tolerability independent of tumor burden, healthy mice received three intravesical instillations of the corresponding control and dFDC‐pGem formulations. Bladder H&E staining showed preserved urothelial and bladder‐wall architecture without evident erosion, edema, hemorrhage, or inflammatory‐cell infiltration across the treatment groups (Figure S31). Independent histopathological grading using the Hopkins 0–5 inflammation scale assigned all examined bladders a score of 0, indicating no detectable local inflammatory injury under the conditions tested. Consistently, neither major‐organ histology nor serum biochemical markers of hepatic and renal function (ALT, AST, BUN, creatinine; Figure S32) showed any treatment‐related abnormalities.

An independent validation cohort with five mice per group reproduced the overall treatment hierarchy observed in the original experiment (Figure S33). Intermediate formulations occasionally produced marked but transient tumor regression, whereas only 2S4F consistently maintained complete suppression throughout the 60‐day observation period.

Together, these results demonstrate that ratio‐engineered dFDCs provide a safe and highly effective platform for intravesical delivery of polymeric gemcitabine, with the 2S4F configuration achieving the optimal balance of barrier traversal and tumor‐cell engagement, resulting in complete remission and durable protection against recurrence.

## 3 Discussion

Multifunctional nanomedicines are frequently assembled through additive incorporation of independent modules, yet successful biological transport often requires balancing orthogonal functions rather than maximizing individual properties. Intravesical delivery provides a particularly stringent example that requires coordinated passage through mucus, selective tumor engagement, and efficient intracellular drug action. Here, we show that a DNA framework drug carrier can coordinate these otherwise decoupled requirements into a cooperative transport–targeting–delivery cascade through precise S/F ratio engineering on its surface, enabling efficient bladder‐tumor delivery at ultralow drug doses.

The translational relevance of this design is supported by patient‐derived evidence showing elevated PTK7 expression in a substantial subset of bladder cancers and by recent first‐in‐human intravesical Sgc8‐based PET imaging, demonstrating that the Sgc8–PTK7 axis is clinically accessible [28]. At the same time, the physiological barrier environment is more difficult to reproduce experimentally. The RT4‐derived GAG‐associated epithelial model used in the transwell assays provides a standardized system for comparing formulation‐dependent transport, but it does not fully recapitulate the spatial heterogeneity and structural complexity of native bladder mucosa. Future validation using intact bladder explants or patient‐/large‐animal‐derived tissues with preserved luminal surfaces will therefore be important for further assessing transport across physiologically remodeled urothelial barriers.

The in vitro analyses—from MST‐based mobility measurements to transwell migration/partitioning, cellular uptake, and post‐translocation killing—showed that framework‐based presentation conferred clear functional advantages over the corresponding simple oligo modifications. Within the dFDC series, these functional gains showed a strongly nonlinear dependence on S/F ratio, with intermediate compositions, most notably 2S4F, achieving a balanced profile of enhanced mucosal transport, efficient PTK7‐mediated uptake, reduced lysosomal sequestration, and potent cytosolic cargo delivery. This emergent synergy highlights a central conceptual insight: successful navigation of hierarchical biological barriers depends on functional coordination rather than maximal enhancement of individual properties. These principles translated directly into in‐vivo outcomes. Under an ultralow monomer‐equivalent dose (∼1/1500 of the clinical formulation), ratio‐engineered dFDCs reproduced the functional hierarchy observed in vitro, with 2S4F achieving rapid tumor clearance during the dosing period and durable remission without recurrence. The superiority of 2S4F‐dFDC over high‐dose free gemcitabine reflects not only its balanced mucosal transport and tumor targeting properties, but also the nanoscale drug‐condensation advantage inherent to the DNA framework. By co‐delivering 60 locally concentrated polyGem strands at each tumor‐contact event, the carrier unifies efficient traversal, precise engagement, and potent payload release into a single therapeutic mechanism that free monomers cannot emulate.

Beyond efficacy, the modularity and structural fidelity of the dFDC architecture provide a broadly extensible platform. In addition to alternative polyhedral geometries and sizes [35], the addressable surface can accommodate diverse functional modules—targeting ligands, mucopenetrating motifs, immunomodulatory epitopes, or biochemical logic gates—while the inner cavity can load diverse nucleic acids, chemotherapeutics, radiosensitizers, or enzyme/prodrug systems. Given that the current pGem payload does not saturate the framework's loading capacity, further increases in per‐particle drug density and multi‐cargo configurations are readily achievable. This modularity also offers a clear route to adapting dFDCs for more challenging clinical contexts—including heterogeneous, deeply invasive, or BCG‐unresponsive bladder cancer—through tailored combinations of functional modules and drugs.

Closely related DNA frameworks bearing the same fluorocarbon and Sgc8 surface modules previously showed low intrinsic cytotoxicity in the absence of therapeutic cargo [30]. Consistently, repeated intravesical administration in the present study produced no detectable hepatic, renal, or local bladder toxicity under the conditions examined, although more extensive repeat‐dose and long‐term toxicological evaluation will be required for clinical translation. In addition, because the fluorocarbon module is derived from an 8:2 fluorotelomer C8F17 moiety, the environmental persistence and degradation behavior of the fluorinated component remain to be established. Future development should therefore include dedicated environmental‐risk assessment and controlled disposal of fluorinated waste.

Overall, this work establishes ratio‐engineered DNA frameworks as a general strategy for programming multistep biological transport. By converting the complexities of the bladder environment into programmable design parameters, dFDCs provide a generalizable blueprint for safe, ultralow‐dose, mechanism‐driven intravesical therapy. As DNA nanoarchitectures continue to advance in stability [36], manufacturability [37], and modularity [38], we anticipate that this design principle will prove broadly applicable to next‐generation intravesical therapies and to other organ systems where layered biological barriers have long limited therapeutic performance.

## Author Contributions

D.L. performed most of the experiments, analyzed the data, arranged the figures, and prepared the manuscript. X.C. performed most of the cell culturing experiments, participated in the animal experiments, and data analysis. H.B. synthesized all the F‐C modified oligos. Q.X., G.H., and Y.Y. jointly conceived and supervised the project, interpreted the data, and finalized the manuscript. All authors reviewed and approved the manuscript.

## Conflicts of Interest

The authors declare no conflicts of interest.

## Supporting information




**Supporting File 1**: advs78719‐sup‐0001‐SuppMat.docx.

**Supporting File 2**: advs78719‐sup‐0002‐SuppMat.docx.

**Supporting File 3**: advs78719‐sup‐0003‐SuppMat.docx.

## Data Availability

The data that support the findings of this study are available in the supplementary material of this article.
