# Supplementary material for: Ligand‐Ratio–Tuned DNA Nanocarriers Overcome Bladder Barriers for Ultralow‐Dose Intravesical Therapy
Source: Adv Sci (Weinh). 2026 Nov 9:e78719. Online ahead of print. doi: 10.1002/advs.78719 (PMC13748771; doi:10.1002/advs.78719)
Supplement: Supplementary file 1 [file ADVS-9999-e78719-s001.docx]

**Supplementary Information**

**Ligand-ratio–tuned DNA nanocarriers overcome bladder barriers for ultralow-dose intravesical therapy**

Dunfang Liu, Xiao Chen, Hongliang Bao, Qian Xia, Gang Huang, and Yang Yang

**Contents**

Abbreviations and construct nomenclature	1

Methods	1

Supplementary Figures	11

Figure S1.	11

Figure S2.	12

Figure S3.	13

Figure S4.	14

Figure S5.	15

Figure S6.	16

Figure S7.	17

Figure S8.	18

Figure S9.	19

Figure S10.	20

Figure S11.	21

Figure S12.	21

Figure S13.	22

Figure S14.	23

Figure S15.	24

Figure S16.	24

Figure S17.	25

Figure S18.	26

Figure S19.	26

Figure S20.	27

Figure S21.	28

Figure S22.	29

Figure S23.	30

Figure S24.	31

Figure S25.	32

Figure S26.	33

Figure S27.	35

Figure S28.	36

Figure S29.	37

Figure S30.	38

Figure S31.	38

Figure S32.	40

Supplementary Tables	41

[Table S1.	41](#_Toc91778425)

[Table S2.	42](#_Toc91778425)

**Abbreviations and construct nomenclature**

| Abbreviation | Definition |
| --- | --- |
| dFDC | DNA framework drug carrier |
| S | Sgc8 aptamer |
| F | fluorocarbon-modified oligo |
| N | unmodified dFDC |
| nS / nF | nominal n/6 S- or F-modified dFDC |
| mSnF | dual-modified dFDC with nominal m/6 S and n/6 F |
| pGem | polymeric gemcitabine |
| GAG | glycosaminoglycan |
| MST | microscale thermophoresis |
| AGE | agarose gel electrophoresis |
| nsTEM | negative-stain transmission electron microscopy |
| PTK7 | protein tyrosine kinase 7 |
| T/B | tumor-to-bladder ratio |
| LD / HD | low-/high-dose free gemcitabine |

**Methods**

**Materials and Regents**

The p7560 scaffold DNA was purchased from Integrated DNA Technologies (IDT) and used without further purification. All DNA staples and fluorescently labeled DNA (Cy5) was obtained from Sangon Biotech (Shanghai, China). 1H,1H,2H,2H-Perfluoro-1-decanol was obtained from Sigma. N, Ndiisopropylethylamine, dichloromethane, 1,1,2,2-Tetrahydroperfluoro dodecanol and 2-cyanoethyl-N,N-diisopropylchlorophosphoramidite, were purchased from J&K Scientific. The reagent for DNA synthesis was obtained from Kroma BioScience (Suzhou China), Wuhu Stong Biotech (Wuhu, China), and GenePoly (Suzhou, China). Nucleic acid stain TS-Gel Red and 1kb DNA ladder were purchased from Beijing Qingke Biotechnology. The Amicon Centrifugal Filters (MWCO 30kDa) was purchased from Merck Millipore. All other chemicals or materials were purchased from Sigma–Aldrich and used as received unless stated otherwise. Cell Counting Kit-8 was purchased from Epizyme Biotech. Hoechst-33342 and LysoTracker Green were purchased from Thermo Fisher Scientific (Shanghai, China). Cell culture reagents including Roswell Park Memorial Institute 1640 (RPMI 1640), McCoy's 5A Medium, Dulbecco's Modified Eagle Medium (DMEM), fetal bovine serm (FBS) and Phosphate buffer saline (PBS) were purchased from Gibco (Thermo Fisher Scientific, MA, USA). Amersham NAP-10 columns were purchased from Cytiva Life Sciences (Cytiva, Marlborough, MA, USA). Double distilled water was purified using a Millipore simplicity system (Millipore, Bedford). All other chemicals were of analytical grade and used without further purification.

**Cell culture and animals**

All cell lines used in this study were obtained from Fuheng Biology (Shanghai, China), including 5637, T24, luciferase-labeled 5637, and GFP-labeled 5637 cells. Cells were used without further authentication after purchase and were routinely tested to be free of mycoplasma contamination. All cultures were maintained at 37 °C in a humidified atmosphere containing 5% CO_2_.

Female BALB/c nude mice (4 ~ 5weeks old) were purchased from Cyagen Guangzhou Biosciences, China). The mice were housed in pathogen-free and ventilated cages and allowed free access to food and autoclaved water in a 12 h light/dark cycle, with room temperature at 20 ~ 22 °C and humidity between 45 and 65%. The animals were maintained in accordance with the guidelines of the Shanghai Medical Experimental Animal Care (AD20240445).

**Self-assembly and purification of DNA Framework**

The single-stranded DNA scaffold p7560 was mixed with requested staple strands at a molar ratio of 1:10 in 1×TE-Mg^2+^ buffer (25mM Tris EDTA, 10mM MgCl_2_, ph 7.4). Thermal annealing procedures for each structure were optimized as follows: 75 °C for 5 min, from 65 to 4 °C for 36 min/−1 °C. The successfully assembled dFDC were purified using rate-zonal centrifugation (50 krpm, 1 hour) through a glycerol gradient (15 %−45 %, v/v). The fractionated layers containing the well-assembled structures were identified by agarose gel electrophoresis (AGE), combined and concentrated through an Amicon Ultracentrifugal filter (Millipore 30 kDa, 5min at 7500 rpm and washed three times using 1×TE-Mg^2+^ buffer).

**Synthesis and purification of FC-modified oligonucleotides**

The synthesis of the FC phosphoramidite monomer followed a patent protocol. Briefly, 1H,1H,2H,2H - Perfluoro-1-octanol (1.49 g, 3.2 mmol) was mixed with dry N,N-diisopropylethylamine (2.3 mL, 5.6 mmol) and 2-cyanoethyl-N,N diisopropyl-chlorophosphoramidite (1 g, 4.2 mmol) in dry dichloromethane (20 mL). The resulting mixture was stirred at room temperature for 2 h. Subsequently, the reaction was halted by adding a 5% NaHCO_3_ aqueous solution (50 mL). After the addition of dichloromethane (30 mL), the aqueous layer underwent three extractions with dichloromethane (50 mL each time). The combined organic layers were dried over anhydrous Na_2_SO_4_, and the solvent was removed under reduced pressure. The obtained residue was co-evaporated with 5 mL of acetonitrile three times and directly utilized for DNA synthesis without further purification.

FC-modified oligonucleotides (F-z’) were synthesized on an automated DNA/RNA synthesizer at a 1 μmol scale through solid-phase phosphoramidite chemistry. The DNA synthesizer was used as recommended by the manufacturer, except that the coupling time was extended to 15 minutes for the incorporation of FC. Once the synthesis was completed, the oligomer was cleaved from the solid support and deprotected by ammonia solution at room temperature overnight. FC-modified oligonucleotide was purified by reverse-phase high-performance liquid chromatography (RP-HPLC). A linear gradient of aqueous 100 mM triethylammonium acetate (TEAA) and acetonitrile was employed during the purification process. The identity of the FC modified oligomers was verified by liquid chromatography-mass spectrometry (LC-MS) in negative electrospray ionization mode. The calculated mass for F-z’ strands was 5909.8, observed mass was 5909.3.

**Construction of dFDC**

Purified dFDC scaffolds were functionalized through sequential hybridization of inner and outer modification strands. For inner-handle loading, ^64^Cu or Cy5 or or gemcitabine-modified anti-inner-handle strands were added at a 60:1 molar ratio (strand:framework) and incubated at 37 °C for 1 h. For outer-surface modification, Sgc8 (S) or fluorocarbon-modified (F) strands were added in defined molar excess relative to the 30 outer handles. Single-modified constructs (nS or nF) were prepared using n = 1–6 equivalent ratios (corresponding to 5×, 10×, 15×, 20×, 25×, and 30× molar excess relative to framework). Dual-modified constructs (mSnF) were generated by mixing S and F strands at the following input ratios: 5:25, 10:20, 15:15, 20:10, and 25:5 (S:F), followed by incubation at 37 °C for 1 h.

For verification of ratio-controlled outer-surface modification, two model outer-handle binding oligonucleotides labeled with AF488 and AF647, respectively, were hybridized to purified dFDC scaffolds using the same outer-handle hybridization strategy. The two fluorescent strands were added individually or in combination at defined input ratios while maintaining a total of 30 strands per framework, followed by incubation at 37 °C for 1 h.

For verification of inner-cargo loading, Cy5-modified anti-inner-handle strands were added to purified dFDC scaffolds at 0×, 10×, 20×, 30×, 40×, 50×, and 60× molar excess relative to framework, followed by incubation at 37 °C for 1 h.

**Agarose gel electrophoresis analysis (AGE)**

Samples were electrophoresed through 1.5% (w/v) agarose gels at 70 V for 2 h in 0.5×TBE with 10 mM MgCl_2_ for both gel preparation and electrophoresis. Following GelRed staining, nucleic acid bands were visualized under UV illumination using a Bio-Rad ChemDoc Imaging System.

**Transmission electron microscopy (TEM) imaging**

Carbon-coated copper grids were pretreated through a glow-discharge process, a 5 μL droplet of 5 nM sample was deposited on the surface of grid with 1 min, then removed using filter pape; 5 μl 3% uranium acetate was added to wash the grid and blotted away, another 5 μl uranium acetate was used to stain for 1 min, the grid was left to air-dry and preserve at room temperature after removing the excess staining solution. TEM imaging was carried out using a Transmission Electron Microscope (Hitachi-HT7700).

**Microscale Thermophoresis (MST)**

Fluorescence thermophoresis check for dFDC in GAG buffer: diluting the dFDC (60× Cy5) samples in TE buffer to reach the final concentration of 0.5 nM with 50 µL volume. Similarly, diluting the oligo-Cy5 samples to the final concentration of 30 nM to ensure an equivalent number of Cy5 dye in comparison with dFDC. Adding the chondroitin sulfate to all samples with final concentration of 1.188 mg/mL. Dip four Monolith NT.115 Capillary capillaries into each sample assay, place them in the device tray and start tracing their thermophoresis behavior.

**In Vitro Cellular Uptake Capacity**

About 5×10^4^ cells were seeded in 96 well plate, pre-cultured overnight with cell growth medium. After removing the growth medium and washed by PBS, 100 μL fresh medium with FBS containing 5 nM dFDC-Cy5 was added to the wells, incubated for 6 h at 37 °C. Then, the medium was removed and washed by PBS. Trypsin was used to digest the cells in each well, growth medium was added to end the digestion and suspended the cells. Suspended mixture was transferred to new tubes and centrifuged for 5 min at 1500 rpm. Supernatant was removed, and cells were resuspended in PBS. Samples of at least 10000 cells were analyzed in triplicates using a FACS Calibur flow cytometer (BD Biosciences, USA). Flow Jo software was used to quantify the fluorescence positive cells as well as median intensity of the signal.

**Confocal Microscopy and Co-localization analysis**

About 2×10^5^ cells were seed in the 15 mm confocal plates (biosharp), pre-cultured overnight with cell growth medium. After growth medium removal and PBS washes, 100 μL fresh medium without FBS but containing 1 nM Cy5-modified sample was added to the plates, incubated for 6 h at 37 °C. Then, 100× Hoechst dye and lysosome tracker was diluted to 1× by culture medium to stain the nucleus and lysosome. After incubation at 37 °C for 30 min, staining solution was removed, and the cells were washed three times by PBS. Stained cells were covered by fresh medium and imaged via SP8 laser scanning confocal microscope (Leica), and LAS X software (Lecia) was used for image processing.

ImageJ software was used to quantify the colocalization of dFDC and lysosome after cell uptake. The Pearson correlation coefficients (PCCs) values were calculated with Coloc2 mode, in which PCCs can be qualified at the location flowing the drawing lines across the lysosome gathering areas.

**Transwell Assay**

RT4 cells at the density of 2×10^5^ per well were added to cover the topside of a 24-well polyester membrane filter (0.4 μm pore size), whereas the bottom chambers were filled with complete cell culture media. The cells were grown for 2 days.

To evaluate the formation of the RT4 cell barrier, transepithelial electrical resistance (TEER) was measured daily from Day 0 to Day 4 after cell seeding. Cell-free inserts containing the same culture medium were measured in parallel as blank controls. TEER was calculated according to the following equation: TEER (Ω·cm²) = (R_sample − R_blank) × A, where R_sample represents the resistance measured across the RT4 cell-covered insert, R_blank represents the resistance of the cell-free insert, and A represents the membrane surface area.

Then, the above samples were added to the upper chamber, respectively, to reach the concentration of 10 nM in the 100 μL final volume. At different time points, the bottom chamber was tested for fluorescence intensity analysis. A microplate reader was used to measure the fluorescence intensity with excitation wavelength of 640 nm and emission at 670 nm. Each data point was tested in triplicate in three independent experiments.

**Tumor cell cytotoxicity assay in transwell**

RT4 cells were seeded at a density of 2×10^5^ per filter on the topside of a 24-well polyester membrane filter (0.4 μm pore size) and cultured for 2 days. GFP-5637 tumor cells were then seeded on the opposite side of the transwell membrane at a density of 5000 cells per filter, allowed to adhere for 2 h, and were cultured for 1 day. Then, the samples were added to the luminal chamber at the serial final concentration of 0.25/0.5/0.833/2.5/5/8.33 nM-100μL, corresponding to the serial amount of Gem monomer (0.3/0.6/1/3/6/10 μM), incubated for 6 h, untreated cells as the control. Then the samples were removed, washed twice with PBS buffer and added fresh complete cell medium to the luminal chamber for another 48 h. Next, tumor cells were collected, washed and resuspended in flow cytometry staining buffer. Samples were finally analyzed using Becman & CytoFLEX. Raw data were analyzed with Flowjo v10.10.

**Receptor Expression Level of PTK7**

To detect the expression of PTK7 on the surface of the included cell lines by flow cytometry, 1×10^6^ cells were collected and washed with cold phosphate-buffered saline (PBS). The washed cells were resuspended in flow cytometry staining buffer (Invitrogen) and incubated with PTK7 polyclonal antibody (PA5-14909, Invitrogen) on ice for 30 min in the dark, followed by washing with cold PBS. Then the cells were stained with AF488 labeled goat anti rabbit IgG H&L antibody. All the samples were washed again, resuspended in cold PBS, and analyzed using a CytoFLEX Flow Cytometer and FlowJo software.

**Ex vivo Toxicity of dFDC**

Bladder cancer cell lines or SV-HUC-1 cell lines were respectively seeded in a 96-well plate (5000 cells/well) and incubated for 24 h for adherence. Thereafter, 100 μL of RPMI-1640 or DMEM complete cell medium containing different samples at different concertation (0.008/0.025/0.05/0.08/0.25/0.5/0.8/2.5/5/8 nM for 5637 tumor cell and 0.008/0.08/0.8/8 nM for SV-HUC-1 cell line, corresponding to the serial amount of inner Gem 0.01/0.03/0.06/0.1/0.3/0.6/1/3/6/10 μM for 5637 tumor cell and 0.01/0.1/1/10 μM for SV-HUC-1 cell line) were added and incubated for 6 h, untreated cells as the control. Then, the cell medium was removed, and the cells were washed with PBS. After that, 100 μL of fresh complete cell medium were added to the cultured cells for another 48 hours. For CCK-8 analysis, the cell medium was substituted with 100 μL of fresh complete cell medium with 10% CCK-8. After incubating for the appropriate time, cell viability was determined by measuring absorbance at 450 nm in a Synergy 2 Multi-Mode Microplate Reader (Bio-Tek, Winooski, VT, USA).

**Ex vivo stability of dFDC**

Samples were added in the urine with the final concentration of 5 nM. Incubated at 37 °C for 0, 0.5, 1, 2, 4 and 6 h. Then, 1.5% AGE were performed to characterize structure integrity and labeling stability.

**Animal Tumor Models**

Female BLAB/c nude mice (4-6 weeks old) were used following protocols approved by Shanghai Jiaotong University Laboratory Animal Center. The mice under anesthetization were injected with fluc-5637 cells (1 × 10^6^) into their bladder wall via a 0.33 mm × 12.7 mm insulin syringe (Becton, Dickinson, and Company). The D-Luciferin potassium salt (Thermo Fisher Scientific) was used for the bioluminescence imaging (10 mg kg^−1^). 7 days after the cell inoculation, the bioluminescence of bladder could be detected by IVIS Lumina system. Meanwhile, the bladder tumors could also be imaged by ultrasonography, which reconfirmed the establishment of orthotopic bladder cancer model.

**^64^Cu-labeling**

Radiolabelling of the dFDC was achieved by hybridizing ^64^Cu-labelled ssDNA with inner-arms designed on dFDC. Amine group-functionalized ssDNA was mixed with 2-S-(4-isothiocyanatobenzyl)-1,4,7-triazacyclononane-1,4,7-triaceticacid (p-SCN-Bn-NOTA) in dimethyl sulfoxide, and incubated at pH 8.5–9.0 at room temperature for 2 h. NOTA-ssDNA was purified using a NAP-10 desalting column with ddH_2_O as the elution buffer. ^64^Cu (decay half-life: 12.7 h) was produced by a PETtrace cyclotron (GE Healthcare) using the 64Ni(p,n)64Cu reaction. For the radiolabelling of NOTA-ssDNA, ^64^CuCl2 was diluted with sodium acetate (0.1 M, pH 5.5) and mixed with NOTA-ssDNA. Following 30 min incubation at 37 °C with constant shaking, the mixed solution was purified with a NAP-10 column, and a fraction with the highest radioactivity was chosen to hybridize with the dFDC. The 64Cu-ssDNA was tested by radio-TLC system, and the ^64^Cu-dFDC was injected into HPLC system for analysis.

**MRI imaging of tumor**

MRI studies were conducted following the observation plan, using a 9.4T Bruker BioSpec USR 94/30 scanner (Bruker BioSpin). Animals were anaesthetized with isoflurane (4% for induction and 1.5% for maintenance in a 50% O_2_/50% N_2_ mixture) and placed on an MR-compatible cradle. Body temperature and respiration rate were continuously monitored using an MR-compatible monitoring device (model 1030 SA, Small Animal Instruments), interfaced to a small-rodent air heater system to maintain body temperature. After acquiring reference images, a spin-echo-based diffusion-weighted imaging sequence was used to image tumours.

**PET imaging of dFDC in orthotopic murine model of bladder cancer**

On day 8 after tumor induction, mice were randomized into ten groups to botain homogeneous average tumor volume distributions among groups. For imaging, intravesical administration of 15 nM×100 µl of dFDC-^64^Cu, and 0.9 μM×100 µL of oligo-^64^Cu were carried out. For T/B ratio calculation, 5/15/30 nM×100 µL of 2S4F were instilled. Animals were induced with anaesthesia (5% isoflurane in pure oxygen) and placed in a supine position before massaging the abdominal region for bladder evacuation. Immediately afterwards, the corresponding samples were instilled in the bladder through a 24-gauge catheter and incubated for 1 h, before removing the catheter, emptying the bladder and leaving the mice to recover from anaesthesia. After a continuous bladder irrigation with normal saline, animals were re-anaesthetized and 10 min static whole-body PET images acquired, followed by CT scans (Siemens Medical Solutions). PET images were reconstructed using the 3D ordered subset expectation maximization reconstruction algorithm with random, scatter and attenuation corrections. PET-CT images of the same mouse were co-registered and analysed using the image processing tool (Inveon Research Workplace). Plots of concentration of radioactivity versus time were obtained by creating a volume of interest on the upper bladder region using a 3D contour tool and measuring activity (decay corrected) in kilobecquerels. The counts/pixel obtained from the raw PET data were converted to counts/cm^3^ based on a calibration coefficient obtained from phantom PET scans. By assuming the tissue density was approximately 1g/cm^3^, counts/cm^3^ were converted to counts/g, which were then divided by the injected dose to provide the final ROI in %ID/g. After the scans post-injection, mice were enthanized, and organs of interest were harvested to quantify the uptake of ^64^Cu using a gamma counter (PerkinElmer). The tracker accumulation in each organ or tissue was presented as the percentage of the irrigate dose per gram of tissue (%ID/g; n= 2 in each group).

**Ex vivo fluorescence imaging of intravesical retention**

For fluorescence based validation of intravesical retention, orthotopic bladder tumor-bearing mice were intravesically administered Cy5-labeled oligo controls or dFDC variants carrying equivalent amounts of Cy5. Before sample administration, baseline tumor burden was evaluated by bioluminescence imaging. Following intravesical administration, bladders were excised and subjected to ex vivo Cy5 fluorescence imaging. Fluorescence radiance within the bladder region of interest (ROI) was quantified to compare the retention of different formulations (n = 3 mice per group).

**Therapeutic orthotopic bladder tumor experiment**

Seven days after orthotopic tumor cell inoculation, the tumor-bearing female mice were randomly allocated to twelve groups, including saline, free Gem (HD, LD), oligo-pGem (S, F, SF), dFDC-pGem (N, 6S, 6F, 2S4F, 3S3F, and 4S2F). Intravesical instillation was performed by administering 100 μL of each formulation into the bladder and retaining it for 1 h. The LD group received 6.3 μg/mL gemcitabine (monomer-equivalent), and all oligo-pGem and dFDC-pGem groups were normalized to the same gemcitabine-equivalent loading. The HD group received 10 mg/mL free gemcitabine, corresponding to the maximum tolerated intravesical dose in mice. Tumor burden (n = 3 per group) was monitored by bioluminescence imaging and ultrasonography on Days 0, 5, 10, 15, 20, 25, 30, 40, 50, and 60.

Body weight and survival were recorded throughout the 60-day study. Mortality related to tumor progression or metastasis was documented. For histopathological evaluation, two mice per group were sacrificed on Day 11, and major organs (heart, liver, spleen, lung, and kidney) were collected for hematoxylin and eosin (H&E) staining and pathological assessment.

An independent therapeutic experiment was performed with n = 5 mice per group using the same formulation doses, intravesical administration procedure, treatment schedule, imaging time points, and 60-day follow-up protocol.

For survival monitoring, mice were euthanized upon reaching predefined humane endpoints according to the approved animal protocol. Because orthotopic bladder-tumor dimensions could not be reliably assessed by external measurement, humane endpoints were primarily defined by marked wasting with ≥15% body-weight loss, accompanied by reduced food intake and deterioration in general condition. Survival was therefore recorded as time to humane endpoint rather than spontaneous death.

**Ultrasonographic tumor-volume analysis**

For ultrasonography analysis, the acquired 3D datasets were imported into Vevo LAB software (FUJIFILM VisualSonics, Toronto, Canada) and loaded into the 3D analysis module. Tumor volume was measured using the Volume Measurement tool with the Multi-Slice Method and Closed Contour setting. At least five cross-sectional images spanning the entire tumor, including sections at both ends and multiple intermediate sections, were selected for each tumor. Tumor boundaries were manually delineated according to the visible interface between the tumor mass, bladder lumen, and bladder wall on B-mode images, with contour continuity checked against adjacent sections. The software then automatically reconstructed the 3D tumor contour and calculated the tumor volume (mm³). Each tumor dataset was analyzed independently three times, and the mean volume was used for subsequent statistical analysis. The analysis was performed by an independent investigator who was unaware of treatment allocation during tumor segmentation and volume calculation.

**Tumor histological and apoptosis analysis**

For evaluation of treatment induced apoptosis, tumor tissues were collected on Day 11 after three intravesical administrations on Days 0, 5, and 10. Tumor sections from the indicated treatment groups were subjected to cleaved caspase-3 immunofluorescence staining and terminal deoxynucleotidyl transferase dUTP nick-end labeling (TUNEL) staining. For cleaved caspase-3 staining, tumor sections were incubated with an anti-cleaved caspase-3 primary antibody, followed by a fluorescence-labeled secondary antibody and nuclear counterstaining. TUNEL staining was performed using a commercial TUNEL assay kit according to the manufacturer’s instructions, followed by nuclear counterstaining. Fluorescence images were acquired under identical imaging conditions for comparison among treatment groups.

**Bladder histopathological assessment.**

H&E-stained bladder sections were independently evaluated by a pathologist blinded to treatment allocation using the established histopathological grading system reported by Hopkins et al. Bladder inflammation was graded on a 0–5 scale: grade 0, normal bladder tissue; grade 1, focal or multifocal subepithelial inflammatory-cell infiltration; grade 2, diffuse subepithelial inflammatory-cell infiltration accompanied by edema; grade 3, marked subepithelial inflammatory-cell infiltration with necrosis and neutrophils in or on the mucosal epithelium; grade 4, inflammatory-cell infiltration extending into the muscle in addition to the grade-3 features; and grade 5, loss of the surface epithelium with necrosis and full-thickness inflammatory-cell infiltration.

**In vivo toxicity evaluation**

Healthy mice were randomly allocated into four groups (n = 3 per group), including PBS, dFDC-pGem (4S2F, 3S3F, and 2S4F). Intravesical instillation was performed on Days 0, 5, and 10 using the same administration procedure and dose as described in the therapeutic experiment. On Day 11, mice were sacrificed, and the bladder and major organs, including heart, liver, spleen, lung, and kidney, were collected for hematoxylin and eosin (H&E) staining and pathological assessment. Bladder sections were examined for urothelial integrity, inflammatory cell infiltration, edema, hemorrhage, and other apparent pathological alterations.

Blood samples were collected on Day 11 for serum biochemical analysis. Alanine aminotransferase (ALT) and aspartate aminotransferase (AST) were measured for evaluation of hepatic function, while blood urea nitrogen (BUN) and creatinine (CREA) were measured for evaluation of renal function.

**Transcriptomic analysis**

For transcriptomic analysis, untreated control cells (Blank) and cells treated with unmodified dFDC (N), 6S, 6F, 4S2F, 3S3F, or 2S4F were analyzed with three biological replicates per group. Differential gene expression between each dFDC-treated group and the Blank control was analyzed using DESeq2. Genes with q-value < 0.05 and fold change ≥ 2 or ≤ 0.5 were defined as differentially expressed genes.

For targeted analysis of the cGAS-STING and type I interferon-related signaling pathways, log2 fold-change values of representative genes, including cGAS, STING1, TBK1, IRF3, CXCL10, ISG15, IFIT1, MX1, and OAS1, were extracted from each treatment versus Blank comparison and visualized as a heatmap. The pathway-focused heatmap was generated from the complete differential expression dataset without additional significance filtering.

**Statistical analysis**

GraphPad Prism 8.0.1 was used for all data analysis and graph plotting. Data were presented as mean values ± SD. The results were analyzed by the paired or unpaired two-sided Student’s t-test between two groups. One-way and two-way analysis of variance (ANOVA) and multiple comparison was used to determine statistical significance. Exact P values were provided accordingly in the figures or captions. P < 0.05 was used as the threshold for statistical significance. No statistical method was used to predetermine sample size. The exact number of replicates and statistical tests are indicated in the figure legends. Unless otherwise indicated, n represents the number of independent experimental replicates.

**Supplementary Figures**

**Figure S1. Modular architecture, surface-ratio design, and cargo loading of the DNA framework drug carrier (dFDC).** Schematic of the icosahedral dFDC displaying 30 outward-facing z handles for surface functionalization and 60 inward-facing a handles for cargo loading. Surface modules carrying the complementary z′ sequence include the Sgc8 aptamer (S) and fluorocarbon-modified oligonucleotide (F), whereas cargo strands carrying the complementary a′ sequence are used to load Cy5, ^64Cu, or polymeric gemcitabine (pGem) into the framework interior. Single-modified constructs are denoted nS or nF (*n* = 1–6), corresponding to nominal *n*/6 occupation of the 30 available outer handles; dual-modified constructs are denoted mSnF, where *m* + *n* = 6. N denotes the unmodified dFDC. The resulting library therefore spans S-only, F-only, and S/F-dual surface compositions while maintaining a common total outer-handle pool. The lower panel illustrates the corresponding oligonucleotide controls: ss, unmodified single-stranded oligonucleotide; S, Sgc8-modified oligonucleotide; F, fluorocarbon-modified oligonucleotide; and SF, dual Sgc8/fluorocarbon-modified oligonucleotide.

**Figure S2. TEM characterization of dFDCs bearing different S and F surface modifications.** Representative transmission electron microscopy (TEM) images of icosahedral DNA framework drug carriers (dFDCs) bearing Sgc8 aptamer (S), fluorocarbon-modified oligonucleotide (F), combined S/F surface modifications, or no outer-surface modification (N). The frameworks retained intact icosahedral morphology across the full modification library, indicating that surface functionalization did not appreciably alter the overall framework architecture. Scale bar, 50 nm.

**Figure S3. AGE analysis of Cy5-loaded dFDCs bearing different outer-surface S and F modifications.** Native agarose gel electrophoresis (AGE) of dFDCs internally loaded with Cy5-labeled strands (dFDC–Cy5) and bearing Sgc8-only (S; 1S–6S), fluorocarbon-only (F; 1F–6F), or dual S/F surface modifications (1S5F–5S1F). GelRed staining (top) shows intact framework bands across the modification library, with modification-dependent shifts in electrophoretic mobility. Cy5 fluorescence (bottom) confirms successful association of the internally loaded Cy5 strands with the corresponding dFDC constructs. **L**, DNA ladder; **N**, unmodified dFDC; **N (w/o Cy5)**, unmodified dFDC without internal Cy5 loading.

**Figure S4. Experimental validation of ratio-programmable occupation of dFDC outer handles.** AF488- and AF647-labeled anti-z strands that hybridize to the common outer-handle sequence (z) of dFDC were used as fluorescent tracers to evaluate surface-modification stoichiometry. Single-color labeling with increasing nominal input fractions (1/6–6/6) produced approximately linear increases in framework-associated fluorescence for both AF488 and AF647. In the dual-color experiment, complementary AF488/AF647 input fractions generated reciprocal changes in the corresponding fluorescence signals, with strong linear relationships maintained for both channels. Band-associated fluorescence from the gel shown above was quantified using ImageJ as background-subtracted integrated density. AF488 and AF647 intensities in the dual-color plot are referenced to their respective y-axes.

**Figure S5. Programmable loading of inner-handle cargo on dFDC.** Cy5-labeled oligonucleotides complementary to the dFDC inner handles were added at 0, 10, 20, 30, 40, 50, or 60 equivalents per framework and analyzed by native agarose gel electrophoresis. Framework-associated Cy5 fluorescence increased linearly with input strand number, supporting programmable stoichiometric loading through the 60 designed inner handles.

**Figure S6. Native PAGE analysis for constructing oligo-Cy5 control samples and their assembly elements.** Top: Assembly of oligo-Cy5–S and –F controls. Individual strands and intermediate complexes are shown as references, and the final Cy5-S and Cy5-F products appear in lanes 8 and 9. Bottom: Assembly of the dual-functional oligo-Cy5-SF with the fully assembled products appears in lane 6.

 **Figure S7. MST analysis of thermophoretic mobility of dFDC variants and oligonucleotide controls in a GAG-mimicking environment.** Representative microscale thermophoresis (MST) traces of Cy5-labeled dFDCs bearing Sgc8-only (S), fluorocarbon-only (F), or dual S/F surface modifications (solid lines), together with the corresponding Cy5-labeled oligonucleotide controls (S, F, and SF; dashed lines), measured in chondroitin-sulfate–supplemented buffer as a glycosaminoglycan (GAG)–mimicking environment. Enlarged views of the 18–20 s interval highlight the relative thermophoretic-mobility differences among the tested constructs.

**Note:** dFDCs carrying a single Cy5 fluorophore exhibited only marginally faster thermophoretic motion than dFDCs carrying 60 Cy5 fluorophores (top-right panel), consistent with the small difference in total molecular mass. This control indicates that the Cy5 labeling density has only a limited influence on the MST readout under the conditions tested. Accordingly, comparison of the oligo–Cy5 controls with singly labeled unmodified dFDC (N; 1× Cy5) provides an appropriate reference for assessing intrinsic differences in thermophoretic mobility.

**Figure S8. Time-course evaluation of RT4 epithelial barrier formation by transepithelial electrical resistance.** RT4 cells were cultured on Transwell inserts, and TEER was measured daily for 4 days after seeding. TEER increased rapidly during the first 24 h and approached a plateau by Day 2, with only modest additional increases on Days 3–4, indicating establishment of a relatively stable epithelial barrier under the culture conditions used for subsequent translocation assays. Data are presented as mean ± SD (n = 3 independent Transwell inserts).

**Figure S9. Transwell analysis of barrier translocation by dFDC variants and oligonucleotide controls.** Time-dependent fluorescence intensity measured in the bottom chamber after addition of Cy5-labeled DNA framework drug carrier (dFDC) constructs to the upper chamber of an RT4 cell-derived GAG-associated epithelial barrier model. Panels show fluorocarbon-only (F), Sgc8-only (S), dual S/F-modified dFDC variants, and the corresponding Cy5-labeled oligonucleotide controls. RT4 cells were pre-cultured on the insert membrane to establish the GAG-associated epithelial barrier. The measured mean fluorescence intensity (MFI) in the bottom chamber reflects the relative amount of material transported across the cell-derived barrier over 6 h.

**Note:** All variants exhibited a biphasic permeation profile, with an initial rapid transport phase during Stage 1 (0–1 h) followed by a slower, approximately linear regime during Stage 2 (1–6 h). Because construct-dependent differences were more reproducibly resolved during Stage 2, quantitative comparisons of relative barrier-translocation behavior were derived from the Stage-2 linear slopes.

**Figure S10.** **Layer-by-layer distribution of dFDC variants and oligonucleotide controls in the RT4 cell-derived GAG-associated epithelial barrier model.** Percentages of Cy5-labeled DNA framework drug carrier (dFDC) constructs and corresponding oligonucleotide controls detected in the top chamber, membrane-associated middle region, and bottom chamber of the Transwell system 6 h after sample addition. These values represent the relative distribution of material remaining in the donor compartment, retained within the barrier-associated region, or transported into the receiver compartment. Series-level comparisons were performed by two-way ANOVA using composition-matched F-only versus S/F and S-only versus S/F series, as indicated by the brackets in the figure.

**Figure S11. Area-under-curve (AUC) analysis of transwell cytotoxicity for dFDC-pGem variants and oligo-pGem controls.** AUC values (30%–90% viability range) calculated from the dose–response curves of 5637 cells in the two-layer transwell cytotoxicity assay. dFDC-pGem constructs bearing F-only, S-only, and S/F-dual modifications, together with oligo-pGem controls, were compared to evaluate post-translocation killing profiles.

**Figure S12. Direct cellular uptake of dFDC–Cy5 variants under equal-dose incubation in 5637 and T24 tumor cells.** Flow-cytometry quantification of Cy5-labeled dFDC constructs and oligo controls following equal-dose (Cy5-matched) direct incubation with 5637 (top) and T24 (bottom) bladder cancer cells for 6 h. S-bearing and S/F-dual-modified constructs exhibited higher uptake than F-only variants, with consistent ratio-dependent patterns observed across both cell types.

**Figure S13. Lysosomal colocalization of internalized dFDC–Cy5 constructs.**

Confocal fluorescence imaging of 5637 bladder cancer cells after equal-dose direct incubation with Cy5-labeled dFDC variants for 6 h, showing intracellular distribution relative to LysoTracker-labeled lysosomes. Pearson’s correlation coefficients quantify lysosomal colocalization for S-only, F-only, S/F-dual-modified dFDCs and the unmodified control (right). Scale bar: 25 μm.

**Figure S14. Direct cytotoxicity of dFDC-pGem constructs and oligo-pGem controls toward bladder cancer cells.** Dose–response–based cytotoxicity of 5637 (top) and T24 (bottom) bladder cancer cells following equal-dose direct incubation with pGem-loaded dFDC variants for 48 h. IC_50_ values and viability at C_max were derived from flow-cytometry–quantified live/dead discrimination. Oligo-pGem constructs with matched gemcitabine content were included as controls.

**Figure S15. PTK7 surface expression levels across bladder cancer and urothelial cell lines.** Flow-cytometry quantification of membrane PTK7 expression in 5637, T24, RT4, and SV-HUC-1 cells using a primary anti-PTK7 antibody followed by an AF488-conjugated secondary antibody (488 nm channel). Unstained cells served as the blank control. Mean fluorescence intensity (MFI) values reflect relative PTK7 abundance across cell types.

**Figure S16. Differential cytotoxicity of dFDC-2S4F–pGem and ss-pGem in normal urothelial versus bladder cancer cells.** Cell viability of SV-HUC-1 normal urothelial cells and 5637 bladder cancer cells after 48 h direct incubation with increasing concentrations of pGem delivered either by the dFDC-2S4F scaffold or as free ss-pGem. Viability was quantified using a CCK-8 colorimetric assay.

**Figure S17. Transcriptional profiling of the cGAS–STING pathway following dFDC treatment.** Heatmap showing the log_2_ fold changes of representative cGAS–STING pathway genes in cells treated with the indicated dFDC formulations relative to the untreated control. Core pathway genes (CGAS, STING1, TBK1, and IRF3) and representative downstream type-I-interferon/interferon-stimulated genes (CXCL10, ISG15, IFIT1, MX1, and OAS1) are shown. Values within each cell indicate the corresponding log_2_ fold change relative to the untreated control. No coordinated transcriptional induction of the cGAS–STING pathway or its representative downstream interferon-response genes was observed across the tested formulations. RNA-seq was performed with n = 3 independent biological replicates per group.

**Figure S18. Stability of outer and inner modifications of dFDC constructs in mouse urine.** Native agarose gel electrophoresis showing the time-dependent stability of Cy5-labeled outer (left, seq-z’) and inner (right, seq-a’) strands on dFDCs after incubation in freshly collected mouse urine (0–6 h). Bands were visualized using GelRed staining and Cy5 fluorescence to independently track the framework and the associated modification strands.

 **Figure S19. Radiochemical characterization of ^64^Cu-labeled ssDNA controls.**
(**Left**) Radio–TLC profile of ^64^Cu-chelated NOTA–ssDNA (sequence a’) showing clear separation from unchelated free ^64^Cu. (**Right**) Radio–HPLC chromatogram of purified ^64^Cu-ssDNA demonstrating radiochemical purity, with UV absorbance and radioactivity traces shown simultaneously.

**Figure S20. PET/CT imaging and quantitative intravesical retention of ^64^Cu-labeled dFDC variants and oligo controls.** Serial PET/CT images and corresponding time–activity curves show bladder tumor–associated radioactivity (%ID/g) over the imaging period for each formulation. Each treatment included two mice, and those in the first row outlined in red boxes correspond to the animals displayed in **Figure 3C** in the main text.

**Figure S21. Baseline tumor-burden comparability and ex vivo validation of dFDC retention in orthotopic bladder tumors.** Bioluminescence images of luciferase-expressing orthotopic bladder tumors immediately before intravesical administration of the indicated formulations, together with corresponding ROI-based radiance quantification. No significant difference in baseline tumor burden was detected among groups by one-way ANOVA (*p* = 0.7712). Following intravesical administration of Cy5-labeled oligo controls or dFDC variants carrying equivalent Cy5 amounts, bladders were excised and imaged ex vivo. Quantification of Cy5 fluorescence reproduced the composition-dependent retention trend observed by ^64^Cu PET/CT imaging. Data are presented as mean ± SD (n = 3 mice per group).

**Figure S22.** **Bioluminescence monitoring of orthotopic bladder tumors following intravesical treatment.** Complete data set of bioluminescence images from orthotopic bladder tumor–bearing mice collected over a 60-day period after intravesical administration of the indicated formulations. Treatments included free gemcitabine in its monomeric form for the high-dose (HD) and low-dose (LD) groups, and 40-nt pGem delivered by various dFDC constructs or oligo controls (gemcitabine units equivalent to LD). Imaging was conducted at the specified time points to track tumor burden throughout the treatment course. All bioluminescence images were displayed using a uniform lower visualization threshold of 1 × 10^6^ photons·s⁻¹·cm⁻²·sr⁻¹; this value was used for image display only and not as a statistical cutoff for tumor positivity. Each treatment included three mice, and images in the first row outlined in red boxes correspond to the animals displayed in the main **Figure 4B**.

**Figure S23.** **Bioluminescence quantification of intravesically treated mice.** Time-course bioluminescence signals of orthotopic bladder tumors over 60 days following intravesical administration of the indicated formulations. Curves represent individual animals (n = 3 per group). Radiance values (photons/sec/cm²/sr) were extracted at each imaging time point to quantify tumor burden throughout the treatment course.

**Figure S24. Longitudinal ultrasonography monitoring of bladder tumor progression in control-treated mice.**

**Figure S25. Longitudinal ultrasonography monitoring of bladder tumor progression in dFDC-pGem–treated mice.**

Serial ultrasonography images of orthotopic bladder tumors acquired every 5 days over the 60-day study period following intravesical administration of control or dFDC formulations. Tumor boundaries were delineated manually, and corresponding tumor volumes for each mouse are plotted on the right. Each treatment group contained three mice. Images in the first row of each group outlined in red boxes correspond to the ones displayed in the main **Figure 4B**.

**Figure S26. Apoptosis-associated histological analysis of orthotopic bladder tumors after intravesical treatment.** A parallel cohort of orthotopic bladder tumor-bearing mice received three intravesical administrations of the indicated formulations, and one mouse from each treatment group was harvested on Day 11 for histological analysis while the remaining animals continued longitudinal follow-up. Representative images of cleaved caspase-3 immunostaining **(A)** and TUNEL staining **(B)** are shown for non-carrier controls and dFDC-pGem formulations, together with higher-magnification views of the indicated regions. Cleaved caspase-3 and TUNEL signals are shown in red, with nuclei counterstained by DAPI in blue. Quantification of cleaved-caspase-3-positive area and TUNEL-positive area was performed from five non-overlapping fields of view from the examined tumor in each treatment group and is presented as mean ± SD of field-level measurements. These measurements are descriptive and were not treated as independent biological replicates for inferential statistical testing. Scale bars: 100 μm (low magnification) and 50 μm (high magnification).

**Figure S27. Whole-bladder histopathological assessment across the complete therapeutic cohort.** Low-magnification H&E-stained bladder sections from all five mice in each treatment group collected at their respective terminal endpoints. The individual harvest day for each mouse is indicated above the corresponding section. Animals with progressive disease were harvested upon reaching predefined humane endpoints, whereas mice with sustained tumor control were followed to the planned study endpoint. The red-boxed sections indicate samples selected for higher-magnification pathological evaluation in Fig. S28. This complete cohort-level overview enables comparison of overall bladder architecture and residual or recurrent tumor burden across treatment groups. Scale bars, 250 μm.

**Figure S28. Terminal bladder histology and corresponding bioluminescence across treatment groups following intravesical therapy.** Representative terminal bioluminescence images, whole-bladder H&E-stained sections, and corresponding higher-magnification views of selected regions of interest are shown for the indicated treatment groups. The displayed bladder sections correspond to the red-boxed samples in Fig. S27. Because animals in the control and less-effective treatment groups reached predefined humane endpoints at different times, bladders were collected at their respective terminal endpoints, as indicated above each section, whereas animals with sustained tumor control were followed to the planned Day-60 study endpoint. H&E examination showed treatment-dependent differences in residual or recurrent tumor burden, with persistent tumor tissue evident in less-effective groups and preserved bladder architecture with no histologically identifiable residual tumor in the examined 2S4F-pGem section at Day 60. Scale bars, 250 μm for whole-section images and 100 μm for higher-magnification views.

**Figure S29. Longitudinal monitoring of mouse body weight during intravesical treatment.** Body-weight trajectories of mice bearing orthotopic bladder tumors over the 60-day study period following intravesical administration of the indicated formulations. Each treatment group contained three mice.

**Figure S30.** **Histopathological examination of major organs in tumor-bearing mice following intravesical treatment.** H&E–stained sections of major organs—including cardiac tissue, liver, spleen, lung, and kidney—collected from orthotopic bladder tumor–bearing mice on Day 11 after three intravesical administrations on Days 0, 5, and 10 of the indicated formulations. Scale bar, 100 μm.

**Figure S31. Histopathological assessment of local bladder inflammation in healthy mice following intravesical treatment.** H&E-stained bladder sections collected from healthy mice on Day 11 after three intravesical administrations of the indicated formulations on Days 0, 5, and 10. Bladder inflammation was independently evaluated using the Hopkins 0–5 histopathological grading system. No evident treatment-related urothelial injury, edema, hemorrhage, or inflammatory-cell infiltration was observed, and all examined bladder sections were assigned inflammation grade 0. Scale bar, 100 μm. n = 1 mouse per treatment group.

**Figure S32. Serum biochemical analysis of healthy mice after repeated intravesical administration of dFDC-pGem formulations.**

Serum biochemistry indices—including alanine aminotransferase (ALT), aspartate aminotransferase (AST), blood urea nitrogen (BUN), and creatinine (CREA)—measured on Day 11 in healthy mice after three intravesical administrations (Days 0, 5, and 10) of PBS (Control) and dFDC-pGem formulations (4S2F, 3S3F, 2S4F). Data are presented as mean ± SD, n = 3.

**Figure S33. Independent validation of therapeutic efficacy in an expanded cohort.** An independent therapeutic study was performed using five mice per treatment group and the same intravesical dosing regimen and 60-day follow-up schedule as in the original therapeutic experiment. **(A)** Longitudinal bioluminescence images of orthotopic bladder tumor-bearing mice receiving the indicated control or dFDC-pGem formulations. Each row represents an individual animal followed over time; blank positions indicate that the animal had reached the predefined humane endpoint. The dotted vertical line marks the end of the dosing period on Day 30. **(B)** Individual bladder-tumor radiance trajectories corresponding to the animals shown in panel A. Radiance was quantified within the predefined bladder region of interest and is presented for each individual mouse. **(C)** Group-level quantification of tumor bioluminescence for non-carrier controls and dFDC-pGem formulations. Data are presented as mean ± SD (n = 5 mice per group until animals reached the humane endpoint). **(D)** Kaplan–Meier survival curves for the corresponding treatment groups over the 60-day observation period. The overall treatment-response hierarchy closely reproduced that observed in the original cohort.

**Supplementary Tables**

**Table S1. Sequence information of the functional strands**

| Name | Sequence (5’ – 3’) | Length  (nt) |
| --- | --- | --- |
| a' | TAGATGGAGTGTGGTGTGAAG-Cy5/^64^Cu/Gem | 21 |
| linker  (a-z) | CTTCACACCACACTCCATCTA-TTTGAGAGTTAGGAATGT | 39 |
| z' | ACATTCCTAACTCTCAAA | 18 |
| F-z’ | F-C- ACATTCCTAACTCTCAAA | 18 |
| Sgc8-z’ | ATCTAACTGCTGCGCCGCCGGGAAAATACTGTACGGTTAGAACATTCCTAACTCTCAAA | 59 |
| a-Sgc8-z | CTTCACACCACACTCCATCTAATCTAACTGCTGCGCCGCCGGGAAAATACTGTACGGTTAGATTTGAGAGTTAGGAATGT | 81 |
| 20×Gem-a’ | **Gem×20**-TAGATGGAGTGTGGTGTGAAG | 41 |
| 40×Gem-a’ | **Gem×40**-TAGATGGAGTGTGGTGTGAAG | 61 |

**Table S2. Staples strands sequence information of dFDC.**

| dna Framework Drug Carrier (dFDC) | | |
| --- | --- | --- |
| dFDC-Vertex-001 | AGACATTTTTGTCAAATCACCGTACCCCGGTTTTTTTGATAATCAGAAA | 49 |
| dFDC-Vertex-002 | AATTCTTTTTGCGTCTGGCCTAGTATCGGCCTTTTTTCAGGAAGATCGC | 49 |
| dFDC-Vertex-003 | TTTTGTTTTTTTAAAATTCGCA | 22 |
| dFDC-Vertex-004 | AATTATTTTTACCGTTGTAGCGATAGGGTTGATTTTTGTGTTG | 43 |
| dFDC-Vertex-005 | TAGGGTTTTTCGCTGGCAAGTGGAACGGTACGTTTTTCCAGAATCCTGA | 49 |
| dFDC-Vertex-006 | TTCCACCCCCGATTTATTTTTGAGCTTGACGGG | 33 |
| dFDC-Vertex-007 | ATTGCTTTTTTCCTTTTGATATAGATACATTTTTTTTCGCAAATGGTCA | 49 |
| dFDC-Vertex-008 | CCAATTTTTTACTGCGGAATCATCGCGTTTTATTTTTATTCGAGCTTCA | 49 |
| dFDC-Vertex-009 | TTCTATTTTTCTAATAGTAGTA | 22 |
| dFDC-Vertex-010 | AGGCTTTTTTTTTGCAAAAGACCTCATATATTTTTTTTTAAAT | 43 |
| dFDC-Vertex-011 | AAGTTTTTTTTGAGTAACATTAGGAATTACGATTTTTGGCATAGTAAGA | 49 |
| dFDC-Vertex-012 | GCAATGCCAGGGTTTTTTTTTCCCAGTCACGAC | 33 |
| dFDC-Vertex-013 | TAGACTTTTTTTTACAAACAACGCCCTGGAGTTTTTTGACTCTATGATA | 49 |
| dFDC-Vertex-014 | ATAAATTTTTACAGAGGTGAGCTAAAATATCTTTTTTTTAGGAGCACTA | 49 |
| dFDC-Vertex-015 | GGCCTTTTTTTGAATCGGCTGA | 22 |
| dFDC-Vertex-016 | ATGCGTTTTTCGAACTGATAGTTATCCGCTCATTTTTCAATTC | 43 |
| dFDC-Vertex-017 | TCAAATTTTTCTATCGGCCTTCAGAGATAGAATTTTTCCCTTCTGACCT | 49 |
| dFDC-Vertex-018 | CACACGCGTATTGGGCTTTTTGCCAGGGTGGTT | 33 |
| dFDC-Vertex-019 | TCACCTTTTTGTACTCAGGAGCAGCCCTCATATTTTTGTTAGCGTAACG | 49 |
| dFDC-Vertex-020 | GGGTTTTTTTTTGCTCAGTACC | 22 |
| dFDC-Vertex-021 | TTTGCTTTTTTAAACAACTTTGATACCGATAGTTTTTTTGCGCCGACAA | 49 |
| dFDC-Vertex-022 | CATGAGAAGTTTCCATTTTTTTAAACGGGTAAA | 33 |
| dFDC-Vertex-023 | AATAATTTTTATCCTCATTAAAACCTATTATTTTTTTCTGAAA | 43 |
| dFDC-Vertex-024 | ACTAATTTTTAACACTCATCTAAAGAGGACAGTTTTTATGAACGGTGTA | 49 |
| dFDC-Vertex-025 | CCGCCTTTTTGCCAGCATTGATCAACTTTAATTTTTTCATTGTGAATTA | 49 |
| dFDC-Vertex-026 | TTGAGTTTTTCGCTAATATCAG | 22 |
| dFDC-Vertex-027 | CATATTTTTTTCCTGATTATCCAGTACCTTTTTTTTTACATCGGGAGAA | 49 |
| dFDC-Vertex-028 | GCGCAACAGTACATAATTTTTATCAATATATGT | 33 |
| dFDC-Vertex-029 | ATTTTTTTTTGCACCCAGCTAAACATAAAAACTTTTTAGGGAA | 43 |
| dFDC-Vertex-030 | TCCTTTTTTTGAAAACATAGCTTTTTCAAATATTTTTTATTTTAGTTAA | 49 |
| dFDC-Vertex-031 | GGCGTTTTTTTTTAGCGAACCCTTAATTGAGATTTTTATCGCCATATTT | 49 |
| dFDC-Vertex-032 | TAGGATTTTTATCATTACCGCG | 22 |
| dFDC-Vertex-033 | TATAATTTTTAGTACCGACAATCCTTATCATTTTTTTCCAAGAACGGGT | 49 |
| dFDC-Vertex-034 | AAAAGTTTTTAAACGCAAAGAGCCATTTGGGATTTTTATTAGA | 43 |
| dFDC-Vertex-035 | GCCAGTTAGCGTTTGCTTTTTCATCTTTTCATA | 33 |
| dFDC-Vertex-036 | CCCTCTTTTTAGAACCGCCACAACTGGCATGATTTTTTTAAGACTCCTT | 49 |
| dFDC-Vertex-037 | ACTCACATTAATTGGGCGATGGCCCGTTAATA | 32 |
| dFDC-Vertex-038 | AACCGTCTATCATATCGTAAAACT | 24 |
| dFDC-Vertex-039 | GATGAACGGTATCTGTTGGGAAGGAGGCCGG | 31 |
| dFDC-Vertex-040 | CAGGCTGCGCAATCATCTGCCAGT | 24 |
| dFDC-Vertex-041 | TCGTAACCGTGTTGCGTTGCGCTCCAAAAAT | 31 |
| dFDC-Vertex-042 | ATCGGCAAAATTTTATCAACAATATCACGCA | 31 |
| dFDC-Vertex-043 | GAACGCGCCTGTTGGAGGCCGATT | 24 |
| dFDC-Vertex-044 | GGAGCTAAACATAATATGCAACTACGGGCGC | 31 |
| dFDC-Vertex-045 | CAACATGTTTTATAGCACTAAATC | 24 |
| dFDC-Vertex-046 | AGGTGCCGTAATCCCTTATAAAT | 23 |
| dFDC-Vertex-047 | TTCCCAATTCTTTTGAAAATCTCCACCTTTA | 31 |
| dFDC-Vertex-048 | AATTTTTTCACGTTAAGAGGAAGC | 24 |
| dFDC-Vertex-049 | ATCAAAAAGATTCTAAATCGGTTGATAGCGT | 31 |
| dFDC-Vertex-050 | CAGAGCATAAAGTCTACAAAGGCTAGCATCAA | 32 |
| dFDC-Vertex-051 | TTTTTGAGAGATTGCGAACGAGTA | 24 |
| dFDC-Vertex-052 | AGCCTTTATTTTTTGTGTCGAAATTAGCGAG | 31 |
| dFDC-Vertex-053 | TCGCCTGATAAATCAACTAATGCA | 24 |
| dFDC-Vertex-054 | AATACCACATTTATGCGCACGACTATTTTAA | 31 |
| dFDC-Vertex-055 | GGTTGTGAATTCTTGCTGCAAGGC | 24 |
| dFDC-Vertex-056 | AAAGGGGGATGTCAACGCAAGGA | 23 |
| dFDC-Vertex-057 | TCCCGCCAAAATAACCTACCATATGAAGTAT | 31 |
| dFDC-Vertex-058 | TGGAAGGGTTAGTCAGTTGAAAGG | 24 |
| dFDC-Vertex-059 | TGGCAAATCAATGGTGCTTGTTACGCAGAAG | 31 |
| dFDC-Vertex-060 | CTTAAGCTACGTTAAACGGCGGATTATATAGG | 32 |
| dFDC-Vertex-061 | CTCCGTGGGAACTTAACCCCGCTT | 24 |
| dFDC-Vertex-062 | TCGTAATCATGTACCTTTTTAACCGTCTTTA | 31 |
| dFDC-Vertex-063 | GTCTGAGAGACTTCAGTAATAAAA | 24 |
| dFDC-Vertex-064 | AGTCACACGACTCCACGCTGGTTTGAAGAAC | 31 |
| dFDC-Vertex-065 | GCAGCAAGCGGTTCGGCCAACGCG | 24 |
| dFDC-Vertex-066 | CATTAATGAATTGTCATAGCTGT | 23 |
| dFDC-Vertex-067 | CACCAGTACAATGCACCGTAATCAAGGTGTA | 31 |
| dFDC-Vertex-068 | CCATCGATAGCATGGGATCGTCACCATTAGCG | 32 |
| dFDC-Vertex-069 | GGCCGCTTTTGCTTTTCGAGGTGA | 24 |
| dFDC-Vertex-070 | TATCAGCTTGCTTGGTTGCTTTGAATGGGAT | 31 |
| dFDC-Vertex-071 | GGGCGCGTACTATACTACAACGCC | 24 |
| dFDC-Vertex-072 | GCTACAGAGGCTACAGTTAATGC | 23 |
| dFDC-Vertex-073 | TGCCCGTATAATACGTAACAAAGCACAAACA | 31 |
| dFDC-Vertex-074 | TTACCCAAATCATATAAGGGAACC | 24 |
| dFDC-Vertex-075 | GACGGTCAATCTCAAAAATCAGGTAGAATAC | 31 |
| dFDC-Vertex-076 | ATGACCATAAATTTTTGAGGACTA | 24 |
| dFDC-Vertex-077 | CAGAACGAGTATGAAGCCCTTTTTACCAGAG | 31 |
| dFDC-Vertex-078 | AGCTATCTTACCTCCTGAGCAAAAGAGGGTAA | 32 |
| dFDC-Vertex-079 | TCATTTCAATTATGTTTAACGTCA | 24 |
| dFDC-Vertex-080 | TAGATTTTCAGTCTAACGGAACAATTATCAT | 31 |
| dFDC-Vertex-081 | TAATAAAACGAATGTAAATTGGGC | 24 |
| dFDC-Vertex-082 | AAAAATCTAAAGTTCATTTGAATT | 24 |
| dFDC-Vertex-083 | ATTTAACAATTTGAAAATAGCAG | 23 |
| dFDC-Vertex-084 | ACGTCAAAAATTCCGGAATCATAAAGTTGCT | 31 |
| dFDC-Vertex-085 | TAAGAATAAACATATCGCAAGACA | 24 |
| dFDC-Vertex-086 | ATGCAAATCCATCATCACCTTGCTCTTAGAA | 31 |
| dFDC-Vertex-087 | AATTCTTACCATAAAGGGCGACATAACGCGA | 31 |
| dFDC-Vertex-088 | GCGCCAAAGACATTCAGGGATAGCATTCATCG | 32 |
| dFDC-Vertex-089 | CCACCCTCATTTTAGAAACCAATC | 24 |
| dFDC-Vertex-090 | TACGAGCATGTTCATTTTGACGCTAAGAGAA | 31 |
| dFDC-Vertex-091 | TGGAAATACCTATGTATAAAGCCA | 24 |
| dFDC-Vertex-092 | GCGCGTTTTCATGGTGAATTATC | 23 |
| dFDC-Vertex-093 | GCTTTTGATGATTTCGGCATTTTC | 24 |
| dFDC-Vertex-094 | AAACCGAGGAATACAGGAGTGTACGCCGCCA | 31 |
| dFDC-Vertex-095 | CCAGTTACAAAATACGCAATAATA | 24 |
| dFDC-Vertex-096 | TATTCATTAAATTAAACAGCCATAAACATAT | 31 |
| dFDC-Red-001 | CCCAAATCGGACTCCAAGATTGTACACTATTAAAAGGGAGGTTTGGAATGGGGTCG | 56 |
| dFDC-Red-002 | GAGTCTGGAATTAATGTATTTTCAACCGTTCTAATCATATATCAATATAGAGAATC | 56 |
| dFDC-Red-003 | TCTTCGCTCCAGGCAACGGCACCGCTTCTGGTTGGGTAACGCCTGAGTAGCTGGCG | 56 |
| dFDC-Red-004 | GTCACGTTGAGCGAGTGCCATCTGCATCAACAACGACGACTCCTGTAGTGGGCGCA | 56 |
| dFDC-Red-005 | TCGGGAAACTGGGGTGTCAGCTCAGCATAAAGGGCGGTTTAACATACGGCCAGCTG | 56 |
| dFDC-Red-006 | AGTTTCATATTGCTGAGGCGAGAAGATGGCTTTTGACCATAGAGGTCAACAGTTGA | 56 |
| dFDC-Red-007 | ACCCTGTATAGCAAAAAATCATACAGGCAAGGTTTAGAACAGTTTTGCGCGGGAGA | 56 |
| dFDC-Red-008 | TGAATTGTGCCAGGGTCAGTGCCAAGCTTTCTTTTACGCTTTCGACAATGACAATG | 56 |
| dFDC-Red-009 | GATCCCCGGGTTGGTGCATTTCTCCGAACTCTTGAAATTGCCCTAAAAGCTCGAAT | 56 |
| dFDC-Red-010 | ATCCTGTTTCACCGCCAGACGGGCAACAGCTGTAGCCCGAAATACTTCGTTCCGAA | 56 |
| dFDC-Red-011 | GAAAAATATAAACAACCAGTGAGGGTCCAGACGCTGTCTTAAGGTAAATCCTAATT | 56 |
| dFDC-Red-012 | GCTTTCCTCGCCGCGCTCTTTCCAGCGTAACCTTTAGACAGTAGCGGTTCAGAGCG | 56 |
| dFDC-Red-013 | AAGGAGCCACAACTAAAAGCAAACGTGAGAATAACAGCTTCAACAGTTTATCGGTT | 56 |
| dFDC-Red-014 | ATAGTCAGCTTTAAACCGAAGGCAGAATCCCCCTTCAAATGTCATAAACGGATTGC | 56 |
| dFDC-Red-015 | GTTACTTAAAAGTACACCTCGTTTATACCAAGCAACTTTGTTGACCCCGAGGCGCA | 56 |
| dFDC-Red-016 | AAGATTCAGACGTTGGGAACTGGCTCATTATAACGCCAAAATCATTTTGATTTAGG | 56 |
| dFDC-Red-017 | TAAAACAGTGTTTGGAAGCCGTCATCAATATATACAGTAAAGATGATGAAATTGCG | 56 |
| dFDC-Red-018 | ACCCTCAACACGCTGATTCTGTAACCGCCTGCAAGGTTATGCGGTCAGTGGTCAGT | 56 |
| dFDC-Red-019 | TTATATAAATAGTGAAGTGGCACAACGCTGAGGAGAAAACGATAGCTTAAATGCTG | 56 |
| dFDC-Red-020 | ATTATTTACCATTGCAAATTTAGGACAATATTCTGGCCAAGCTGGTAAGATTCACC | 56 |
| dFDC-Red-021 | ATGTACCGTCAGAACCCACTCATCCTCAGAACTCCACAGAGTTTAGTAAGTTTCGT | 56 |
| dFDC-Red-022 | AGCATCGGCGCTGAGGCCACGCATAACCGATATTCATGAGAAGTATTATAGCAACG | 56 |
| dFDC-Red-023 | AAGGCTTGATCTTGACGCTGGCTGACCTTCATGTTTAATTCAGGAGGTAGAAACAC | 56 |
| dFDC-Red-024 | CATCAAGACAAAATCGCTGATTGCTTTGAATATAATGGAATTAGACGGTTAATTAC | 56 |
| dFDC-Red-025 | GTTTAGTACCGTGTGAAAATTTAATGGTTTGACAGTAGGGTCCCGACTGTTATACA | 56 |
| dFDC-Red-026 | GTTTGCCTAGGCCGGATCGAGAGGACCATTACCCCCCTTACAAAATCAAGACTGTA | 56 |
| dFDC-Red-027 | CGGGGTCACCGTTCCACCAGAGCCGCAGTCTCTATTTCGGAGCCAGAAAGTAACAG | 56 |
| dFDC-Red-028 | TAGCCGAAAGCAAGAAAATTGAGTTAAGCCCACCCAAAAGCCTCAGAGCCAGAAGG | 56 |
| dFDC-Red-029 | AAATAAGAAGCGTCTTGCAAACGTCTTACCAAGAGAGAATCAATTTTATTTGTTTA | 56 |
| dFDC-Red-030 | GGGAAGGTATAGAAAATCAGATATTATTTTGTCGACTTGACACCACGGACGGAAAT | 56 |
| dFDC-Ora-z-01 | GGAACCCTAAGAACGTAAGTTTTTCAAGAGTCTAAGCAAATTTGAGAGTTAGGAATGT | 59 |
| dFDC-Ora-z-02 | AGCATGTCAGCTGATAAGCAAACAGATATTCATTTGGGGCTTTGAGAGTTAGGAATGT | 59 |
| dFDC-Ora-z-03 | GATTAAGTGCCGGAAAATTACGCCAATGTGTAGGTAAAGATTTGAGAGTTAGGAATGT | 59 |
| dFDC-Ora-z-04 | TTGAGGGGTTAAATGTGGTGTAGACCAGCTTTTAAGCAACTTTGAGAGTTAGGAATGT | 59 |
| dFDC-Ora-z-05 | CGGGGAGATGTAAAGCCCTGTCGTAGCCGGAATTTTTTAATTTGAGAGTTAGGAATGT | 59 |
| dFDC-Ora-z-06 | GATTTAGTAGAGCTTATCCATATATTTTTGCGAGGAAGGGTTTGAGAGTTAGGAATGT | 59 |
| dFDC-Ora-z-07 | TAAAAATTCAAAGAATATACTTTTCAGAGGGGGTAATAGTTTTGAGAGTTAGGAATGT | 59 |
| dFDC-Ora-z-08 | CTAATCTACAGGAGAACAACCTTACTCGTATTAAATCCTTTTTGAGAGTTAGGAATGT | 59 |
| dFDC-Ora-z-09 | TTCCTGTGGACCTCCTGGTACCGACATCGCCATTAAAAATTTTGAGAGTTAGGAATGT | 59 |
| dFDC-Ora-z-10 | CAAAAGAAATTGCCCTTGATGGTGTTTGATTAGTAATAACTTTGAGAGTTAGGAATGT | 59 |
| dFDC-Ora-z-11 | AATAATCGGACGACAAATATCCCAGTAATTCTCCACCGAGTTTGAGAGTTAGGAATGT | 59 |
| dFDC-Ora-z-12 | AAAGGGATACCACACCCGTTAGAACACGCTGCGACGTTAGTTTGAGAGTTAGGAATGT | 59 |
| dFDC-Ora-z-13 | ATTTCTTAAGAAAGGATTTAATTGTCAGCGGATCCAACAGTTTGAGAGTTAGGAATGT | 59 |
| dFDC-Ora-z-14 | CCGAAAGACTCAAATGAAGCAAAGTATTCATTCCAACCTATTTGAGAGTTAGGAATGT | 59 |
| dFDC-Ora-z-15 | GAACTGACCGCGAAACGCCGGAACCAGCGATTACCAGACGTTTGAGAGTTAGGAATGT | 59 |
| dFDC-Ora-z-16 | GATACATACCAGTCAGTCAGTTGAGCGGAACAAAGAAACCTTTGAGAGTTAGGAATGT | 59 |
| dFDC-Ora-z-17 | GATGAATAATCCTGATAAATAAAGGCAATTCAATAGATAATTTGAGAGTTAGGAATGT | 59 |
| dFDC-Ora-z-18 | AATTGAGGAACAGTGCTCAATATCTATTAACAATCGTCGCTTTGAGAGTTAGGAATGT | 59 |
| dFDC-Ora-z-19 | AAGAACGCAAGAGTCACTATATGTAGATTAAGGACAATATTTTGAGAGTTAGGAATGT | 59 |
| dFDC-Ora-z-20 | GGGACATTACCGCCAGCATTGGCATATCCAGACAGAGGCATTTGAGAGTTAGGAATGT | 59 |
| dFDC-Ora-z-21 | TGTAGCATCGCCACCCTAACACTGCCGCCACCGAGAACAATTTGAGAGTTAGGAATGT | 59 |
| dFDC-Ora-z-22 | AAGACTTTTATTCGGTAACGAGGGAGAGGCTGAGACTCCTTTTGAGAGTTAGGAATGT | 59 |
| dFDC-Ora-z-23 | TTGAGATGCAAGAGTACCCTGACGTGAGGCAGGTCAGACGTTTGAGAGTTAGGAATGT | 59 |
| dFDC-Ora-z-24 | ACCTTTTTCCAAGTTAAAACAAAAGAGAATTAACTGAACATTTGAGAGTTAGGAATGT | 59 |
| dFDC-Ora-z-25 | ACGCTCAAAATACCGATCATATGCTGCGGGAGGTTTTGAATTTGAGAGTTAGGAATGT | 59 |
| dFDC-Ora-z-26 | GGTCATAGCATTAGCATTAGCGTCCCAGTAGCGTTGATATTTTGAGAGTTAGGAATGT | 59 |
| dFDC-Ora-z-27 | CCCCTGCCTGAATTTAGTGCCTTGTGGAAAGCACCACCGGTTTGAGAGTTAGGAATGT | 59 |
| dFDC-Ora-z-28 | ACGGAATAATAATAAGCAAAGTTACCACCACCCTCAGAGCTTTGAGAGTTAGGAATGT | 59 |
| dFDC-Ora-z-29 | CCTTTACACGCTAACGAACGATTTTCCTGAATAGAAAATATTTGAGAGTTAGGAATGT | 59 |
| dFDC-Ora-z-30 | ACCGTCACCACAATCAAAATATTGAATAAGTTAGAAGGCTTTTGAGAGTTAGGAATGT | 59 |
| dFDC-Blue-a-001 | TATTTAAAAAACAGGAACGTCAAAGGGCGAAACTTCACACCACACTCCATCTA | 53 |
| dFDC-Blue-a-002 | GCGAGCTGTTTAGCTACCGGAGAGGGTAGCTACTTCACACCACACTCCATCTA | 53 |
| dFDC-Blue-a-003 | TTCAAAAGCAGCTTTCAGCGCCATTCGCCATTCTTCACACCACACTCCATCTA | 53 |
| dFDC-Blue-a-004 | TCGTCGGTGCGGCCCTAACAACCCGTCGGATTCTTCACACCACACTCCATCTA | 53 |
| dFDC-Blue-a-005 | CCAATAGGTTGTTAAACCTAATGAGTGAGCTACTTCACACCACACTCCATCTA | 53 |
| dFDC-Blue-a-006 | AAGAAAGCGCGAACGTATATAATGCTGTAGCTCTTCACACCACACTCCATCTA | 53 |
| dFDC-Blue-a-007 | AAAATGTTATCCAATATTAAGCAATAAAGCCTCTTCACACCACACTCCATCTA | 53 |
| dFDC-Blue-a-008 | TGCCCGAAACGACGGCGGATGTTCTTCTAAGTCTTCACACCACACTCCATCTA | 53 |
| dFDC-Blue-a-009 | ACCGAACGACATAAATTAATGAGTAAACAGGGCTTCACACCACACTCCATCTA | 53 |
| dFDC-Blue-a-010 | ATCACTTGCACCAGTGTGGCCCTGAGAGAGTTCTTCACACCACACTCCATCTA | 53 |
| dFDC-Blue-a-011 | TAAAAGAGTTTATAATATGTTCAGCTAATGCACTTCACACCACACTCCATCTA | 53 |
| dFDC-Blue-a-012 | TAAATGAATTTTGTCGTTAATGCGCCGCTACACTTCACACCACACTCCATCTA | 53 |
| dFDC-Blue-a-013 | GTCAGGATCAGACCGGAGGAATTGCGAATAATCTTCACACCACACTCCATCTA | 53 |
| dFDC-Blue-a-014 | AAACGAAATGCCACTAAGTTCAGAAAACGAGACTTCACACCACACTCCATCTA | 53 |
| dFDC-Blue-a-015 | ACGATAAAATCATAACACGGAGATTTGTATCACTTCACACCACACTCCATCTA | 53 |
| dFDC-Blue-a-016 | ACCAGAAGGATTTTAAGAAGAAAAATCTACGTCTTCACACCACACTCCATCTA | 53 |
| dFDC-Blue-a-017 | TACATTTGTAGATTAGTTATACTTCTGAATAACTTCACACCACACTCCATCTA | 53 |
| dFDC-Blue-a-018 | TATTAATTAACCTTGCGAGCCAGCAGCAAATGCTTCACACCACACTCCATCTA | 53 |
| dFDC-Blue-a-019 | TTTTGAATAAGAATACTTTATCAAAATCATAGCTTCACACCACACTCCATCTA | 53 |
| dFDC-Blue-a-020 | TTTTCGAGCAACATGTACAGGAAAAACGCTCACTTCACACCACACTCCATCTA | 53 |
| dFDC-Blue-a-021 | GCAAGCCGAAGTACCGGCCACCCTCAGAGCCACTTCACACCACACTCCATCTA | 53 |
| dFDC-Blue-a-022 | CAAGAGAAACCATCGCCTTGCAGGGAGTTAAACTTCACACCACACTCCATCTA | 53 |
| dFDC-Blue-a-023 | ATTGGCCTGCGCATAGAAGAACCGGATATTCACTTCACACCACACTCCATCTA | 53 |
| dFDC-Blue-a-024 | CCCTGAACGGATTCGCCGCAGAGGCGAATTATCTTCACACCACACTCCATCTA | 53 |
| dFDC-Blue-a-025 | GCCTTAAATCTGACCTTAAATAAGGCGTTAAACTTCACACCACACTCCATCTA | 53 |
| dFDC-Blue-a-026 | AAGTATAGAAGTGCCGAACGTCACCAATGAAACTTCACACCACACTCCATCTA | 53 |
| dFDC-Blue-a-027 | AACCGCCTCACCGGAAGTAAGCGTCATACATGCTTCACACCACACTCCATCTA | 53 |
| dFDC-Blue-a-028 | CGCCACCACCCACAAGACAATGAAATAGCAATCTTCACACCACACTCCATCTA | 53 |
| dFDC-Blue-a-029 | CATACATAGTATGTTATCCAGAGCCTAATTTGCTTCACACCACACTCCATCTA | 53 |
| dFDC-Blue-a-030 | TATCCGGTCAAGCAAATTCATATGGTTTACCACTTCACACCACACTCCATCTA | 53 |
| dFDC-Green-a-01 | AGCCCCAATTGTAAACACTACGTGAACCATCACTTCACACCACACTCCATCTA | 53 |
| dFDC-Green-a-02 | ATAACCTGAAAAGGTGTCAGGTCATTGCCTGACTTCACACCACACTCCATCTA | 53 |
| dFDC-Green-a-03 | ACTCCAGCGGTGAGAAGCGATCGGTGCGGGCCCTTCACACCACACTCCATCTA | 53 |
| dFDC-Green-a-04 | CCGACAGTGGGCACGAGACCGTAATGGGATAGCTTCACACCACACTCCATCTA | 53 |
| dFDC-Green-a-05 | TTAAATTTAACGCCATACTGCCCGCTTTCCAGCTTCACACCACACTCCATCTA | 53 |
| dFDC-Green-a-06 | GAAAGCCGGAAAGGAGAAGTACGGTGTCTGGACTTCACACCACACTCCATCTA | 53 |
| dFDC-Green-a-07 | GCATTAACTAGACTGGTACCAAAAACATTATGCTTCACACCACACTCCATCTA | 53 |
| dFDC-Green-a-08 | GTTGTAAACGTTATTATAAGTGTCCTTAGTGCCTTCACACCACACTCCATCTA | 53 |
| dFDC-Green-a-09 | CGCATTTCAACCACCACTCGATAAAGACGGAGCTTCACACCACACTCCATCTA | 53 |
| dFDC-Green-a-10 | TTTCTTTTCCTGAGTAGCCCCAGCAGGCGAAACTTCACACCACACTCCATCTA | 53 |
| dFDC-Green-a-11 | GAAGTGTTTCTGTCCAGATAAGTCCTGAACAACTTCACACCACACTCCATCTA | 53 |
| dFDC-Green-a-12 | ATCTAAAGTTTTCTGTCGAGCACGTATAACGTCTTCACACCACACTCCATCTA | 53 |
| dFDC-Green-a-13 | AAGCGAACTAGAGAGTAAAAAAAAGGCTCCAACTTCACACCACACTCCATCTA | 53 |
| dFDC-Green-a-14 | ATACGTAAGAGGCAAACTTTACCCTGACTATTCTTCACACCACACTCCATCTA | 53 |
| dFDC-Green-a-15 | GCAACACTAACCAAAACCGCGACCTGCTCCATCTTCACACCACACTCCATCTA | 53 |
| dFDC-Green-a-16 | CCTTATGCGAGCGGAACATTATTACAGGTAGACTTCACACCACACTCCATCTA | 53 |
| dFDC-Green-a-17 | ACAACTAAAGGATTTACAAAATTATTTGCACGCTTCACACCACACTCCATCTA | 53 |
| dFDC-Green-a-18 | GAGTGAATAATTTTCCGAACCTCAAATATCAACTTCACACCACACTCCATCTA | 53 |
| dFDC-Green-a-19 | GAAAGCGTGGCTATTATCCGGCTTAGGTTGGGCTTCACACCACACTCCATCTA | 53 |
| dFDC-Green-a-20 | AACAACGCCCAGTAATCAATCGTCTGAAATGGCTTCACACCACACTCCATCTA | 53 |
| dFDC-Green-a-21 | ATTAAACCTTTTTATTAGCCCAATAGGAACCCCTTCACACCACACTCCATCTA | 53 |
| dFDC-Green-a-22 | TGACAACAGGATTAGGCTCAGCAGCGAAAGACCTTCACACCACACTCCATCTA | 53 |
| dFDC-Green-a-23 | CAGACCAGTGATATTCTGCTCATTCAGTGAATCTTCACACCACACTCCATCTA | 53 |
| dFDC-Green-a-24 | ACAATAACAAAGTCAGAAGATGATGAAACAAACTTCACACCACACTCCATCTA | 53 |
| dFDC-Green-a-25 | TTTCATCTTCAAGATTTTACTAGAAAAAGCCTCTTCACACCACACTCCATCTA | 53 |
| dFDC-Green-a-26 | AGGCGGATCCCGGAATGTAGCGACAGAATCAACTTCACACCACACTCCATCTA | 53 |
| dFDC-Green-a-27 | ATCAAAATCCCTCAGATGGTAATAAGTTTTAACTTCACACCACACTCCATCTA | 53 |
| dFDC-Green-a-28 | AGAGATAAGAACCACCAAGAAAAGTAAGCAGACTTCACACCACACTCCATCTA | 53 |
| dFDC-Green-a-29 | ATTACGCAAAGGTGGCTTATTTATCCCAATCCCTTCACACCACACTCCATCTA | 53 |
| dFDC-Green-a-30 | CCCAATAGATTCTAAGTCAACCGATTGAGGGACTTCACACCACACTCCATCTA | 53 |
