# Supplementary material for: Ligand‐Ratio–Tuned DNA Nanocarriers Overcome Bladder Barriers for Ultralow‐Dose Intravesical Therapy
Source: Adv Sci (Weinh). 2026 Nov 9:e78719. Online ahead of print. doi: 10.1002/advs.78719 (PMC13748771; doi:10.1002/advs.78719)
Supplement: Supplementary file 2 [file ADVS-9999-e78719-s002.docx]

**Extended data 2:** Complete dataset from the direct cytotoxicity assay evaluating dFDC-pGem and oligo-pGem treatments against 5637 and T24 bladder cancer cells. Cell viability was quantified by CCK-8 analysis following 48 h incubation.

**Extended figure 3:** Dose–response curves in the direct cytotoxicity assay against 5637 cells.

**Extended Table 3:** Summary of IC_50_ values of monomer-equivalent gemcitabine for all formulations in the direct cytotoxicity assay against 5637 cells.

| IC_50_ of monomer-equivalent Gem (μM) in dFDC-pGem | | | | | | | |
| --- | --- | --- | --- | --- | --- | --- | --- |
| 1S | 0.66 | | **1F** | 0.87 | **1S5F** | | 0.42 |
| 2S | 0.51 | | **2F** | 0.78 | **2S4F** | | 0.34 |
| 3S | 0.44 | | **3F** | 0.71 | **3S3F** | | 0.35 |
| 4S | 0.57 | | **4F** | 0.68 | **4S2F** | | 0.43 |
| 5S | 0.56 | | **5F** | 0.74 | **5S1F** | | 0.43 |
| 6S | 0.91 | | **6F** | 0.76 | **N** | | 1.12 |
| IC_50_ of monomer-equivalent Gem (μM) in oligo-pGem | | | | | | | |
| S | | 0.91 | | **SF** | | 0.75 | |
| F | | 0.83 | | **ss** | | 1.02 | |

**Extended Figure 4:** Dose–response curves in the direct cytotoxicity assay against T24 cells

**Extended Table 4:** Calculated IC_50_ values of monomer-equivalent gemcitabine for all formulations in the direct cytotoxicity assay against T24 cells.

| IC_50_ of monomer-equivalent Gem (μM) in dFDC-pGem | | | | | | | |
| --- | --- | --- | --- | --- | --- | --- | --- |
| 1S | 0.33 | | **1F** | 0.41 | **1S5F** | | 0.22 |
| 2S | 0.28 | | **2F** | 0.36 | **2S4F** | | 0.19 |
| 3S | 0.24 | | **3F** | 0.36 | **3S3F** | | 0.2 |
| 4S | 0.28 | | **4F** | 0.36 | **4S2F** | | 0.23 |
| 5S | 0.33 | | **5F** | 0.4 | **5S1F** | | 0.23 |
| 6S | 0.34 | | **6F** | 0.43 | **N** | | 0.87 |
| IC_50_ of monomer-equivalent Gem (μM) in oligo-pGem | | | | | | | |
| S | | 0.7 | | **SF** | | 0.56 | |
| F | | 0.76 | | **ss** | | 0.76 | |
