# Supplementary material for: Ligand‐Ratio–Tuned DNA Nanocarriers Overcome Bladder Barriers for Ultralow‐Dose Intravesical Therapy
Source: Adv Sci (Weinh). 2026 Nov 9:e78719. Online ahead of print. doi: 10.1002/advs.78719 (PMC13748771; doi:10.1002/advs.78719)
Supplement: Supplementary file 3 [file ADVS-9999-e78719-s003.docx]

**Extended data 1:** Complete dataset from the transwell cytotoxicity assay evaluating dFDC-pGem and oligo-pGem treatments against 5637 bladder tumor cells, including the full dose–response curves and corresponding flow-cytometry quantification of apoptotic fractions.

**Extended Figure 1.** Flow-cytometry analysis of the transwell cytotoxicity assay, showing apoptotic profiles of 5637 cells collected from the bottom chamber across all treatment groups.

**Extended Table 1.** Summary of apoptotic counts of 5637 cells retrieved from the bottom chamber in the transwell cytotoxicity assay and analyzed by flow-cytometry.

| Conc. (μM) | 0 | 0.01 | 0.03 | 0.1 | 0.3 | 1 | 3 | 10 |
| --- | --- | --- | --- | --- | --- | --- | --- | --- |
| 1F | 7974 | 7545 | 5536 | 5308 | 4073 | 3442 | 1326 | 1250 |
| 2F | 7812 | 7468 | 5386 | 4865 | 4837 | 2862 | 1341 | 1068 |
| 3F | 7671 | 7050 | 4997 | 4545 | 4053 | 3093 | 1462 | 1020 |
| 4F | 7979 | 7494 | 5337 | 4767 | 4225 | 3182 | 1387 | 1243 |
| 5F | 7054 | 6800 | 5176 | 4790 | 3932 | 2916 | 1282 | 1195 |
| 6F | 6196 | 5901 | 4074 | 3932 | 3515 | 2212 | 1120 | 938 |
| 1S5F | 7509 | 6410 | 5869 | 3910 | 3365 | 2557 | 1871 | 1076 |
| 2S4F | 6978 | 6663 | 4670 | 4108 | 2207 | 1254 | 784 | 723 |
| 3S3F | 7859 | 7354 | 5617 | 5160 | 3842 | 1294 | 831 | 714 |
| 4S2F | 9463 | 8754 | 6503 | 5487 | 4886 | 3162 | 1767 | 1009 |
| 5S1F | 6502 | 6067 | 4652 | 3829 | 3515 | 2513 | 1341 | 968 |
| 1S | 7069 | 6983 | 4972 | 4379 | 3466 | 3278 | 1969 | 1550 |
| 2S | 6603 | 6403 | 4772 | 3864 | 3061 | 2735 | 2286 | 1707 |
| 3S | 7072 | 6911 | 5461 | 4162 | 4042 | 2554 | 2465 | 1546 |
| 4S | 7691 | 7415 | 5625 | 4734 | 4503 | 2666 | 2261 | 1548 |
| 5S | 7701 | 7476 | 5848 | 5369 | 4108 | 3348 | 2477 | 1840 |
| 6S | 6025 | 5731 | 5320 | 4273 | 3804 | 3434 | 1243 | 1165 |
| N | 6590 | 6337 | 5919 | 5024 | 4373 | 3589 | 2427 | 2051 |

| Conc.  (μM) | 0 | 0.01 | 0.03 | 0.1 | 0.3 | 1 | 3 | 10 |
| --- | --- | --- | --- | --- | --- | --- | --- | --- |
| S | 7898 | 7496 | 5822 | 4656 | 4485 | 3806 | 2475 | 1989 |
| F | 6852 | 6784 | 5933 | 4480 | 3913 | 3553 | 2162 | 1616 |
| SF | 7132 | 6774 | 5031 | 4769 | 3517 | 3466 | 2196 | 1293 |
| ss | 6471 | 6341 | 5738 | 4755 | 3908 | 3245 | 2253 | 1659 |

 **Extended Figure 2.** Dose-response curves of each group in the transwell cytotoxicity assay upon calculated cell fractions from flow-cytometry analysis.

**Extended Table 2.** Summary of IC_50_ values of monomer-equivalent gemcitabine for all formulations in the transwell cytotoxicity assay against 5637 cells..

| IC_50_ of monomer-equivalent Gem (μM) in dFDC-pGem | | | | | | | |
| --- | --- | --- | --- | --- | --- | --- | --- |
| 1S | 0.39 | | **1F** | 0.41 | **1S5F** | | 0.29 |
| 2S | 0.35 | | **2F** | 0.49 | **2S4F** | | 0.14 |
| 3S | 0.28 | | **3F** | 0.49 | **3S3F** | | 0.19 |
| 4S | 0.3 | | **4F** | 0.47 | **4S2F** | | 0.28 |
| 5S | 0.34 | | **5F** | 0.59 | **5S1F** | | 0.32 |
| 6S | 0.34 | | **6F** | 0.76 | **N** | | 1.37 |
| IC_50_ monomer-equivalent Gem (μM) in oligo-pGem | | | | | | | |
| S | | 0.71 | | **SF** | | 0.45 | |
| F | | 0.69 | | **ss** | | 0.97 | |
